# Disruption of the Homogentisate Solanesyltransferase Gene Results in Albino and Dwarf Phenotypes and Root, Trichome and Stomata Defects in *Arabidopsis thaliana*

**DOI:** 10.1371/journal.pone.0094031

**Published:** 2014-04-17

**Authors:** Yuehui Chao, Junmei Kang, Tiejun Zhang, Qingchuan Yang, Margaret Yvonne Gruber, Yan Sun

**Affiliations:** 1 Institute of Animal Science, Chinese Academy of Agricultural Sciences, Beijing, People's Republic of China; 2 Saskatoon Research Centre, Agriculture and Agri-Food Canada, Saskatoon, Saskatchewan, Canada; 3 College of Animal Science and Technology, China Agriculture University, Beijing, People's Republic of China; Agriculture and Agri-Food Canada, Canada

## Abstract

Homogentisate solanesyltransferase (HST) plays an important role in plastoquinone (PQ) biosynthesis and acts as the electron acceptor in the carotenoids and abscisic acid (ABA) biosynthesis pathways. We isolated and identified a T-DNA insertion mutant of the *HST* gene that displayed the albino and dwarf phenotypes. PCR analyses and functional complementation also confirmed that the mutant phenotypes were caused by disruption of the *HST* gene. The mutants also had some developmental defects, including trichome development and stomata closure defects. Chloroplast development was also arrested and chlorophyll (Chl) was almost absent. Developmental defects in the chloroplasts were consistent with the SDS-PAGE result and the RNAi transgenic phenotype. Exogenous gibberellin (GA) could partially rescue the dwarf phenotype and the root development defects and exogenous ABA could rescue the stomata closure defects. Further analysis showed that ABA and GA levels were both very low in the *pds2-1* mutants, which suggested that biosynthesis inhibition by GAs and ABA contributed to the *pds2-1* mutants' phenotypes. An early flowering phenotype was found in *pds2-1* mutants, which showed that disruption of the *HST* gene promoted flowering by partially regulating plant hormones. RNA-sequencing showed that disruption of the *HST* gene resulted in expression changes to many of the genes involved in flowering time regulation and in the biosynthesis of PQ, Chl, GAs, ABA and carotenoids. These results suggest that *HST* is essential for chloroplast development, hormone biosynthesis, pigment accumulation and plant development.

## Introduction

**Figure 1:**
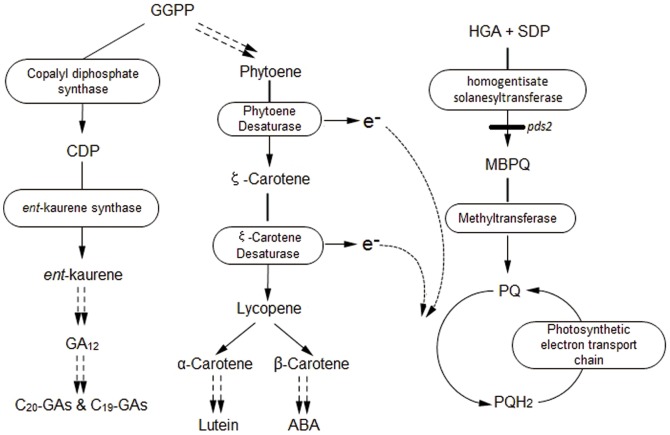
GA, carotenoid, ABA and PQ biosynthesis pathways in plants. PQ acts as an intermediate electron carrier for phytoene, ζ-carotenoid desaturation enzymes and the photosynthetic electron transport chain. GGPP: geranyl geranyl diphosphate; CDP: copalyl diphosphate; e^-^: electron; HGA: homogentisic acid; SDP: solanesyl diphosphate; *pds2-1*: HST mutant; MBPQ: 2-demethyl pastoquinonol; PQH_2_: plastoquinol.

HST is an important enzyme that catalyzes the condensation of the tyrosine-derived aromatic compound, homogentisate (HGA), with the isoprenoid, *all-trans*-nonaphenyl diphosphate solanesyl diphosphate, to form 2-Me-6-solanesyl-1,4-benzoquinol (also known as 2-demethylplastoquinol-9) [1]. This branch-point compound directly leads to the biosynthesis of either vitamin E or the photosystem II (PSII) mobile electron transport co-factor, PQ [2], [3]. Indirectly, it also links a number of other diverse, important metabolic pathways, including carotenoid biosynthesis, which uses PQ as a co-factor for phytoene desaturase, and ABA biosynthesis, which is derived from the breakdown of carotenoids [4]–[6]. Consequently, HST is likely to affect plant growth and development. While the homogentisate and solanesyl pathways are critical for forming prenylated electron carrier molecules, such as PQ in chloroplasts and ubiquinone in mitochondria (Figure 1, modified from Motohashi et al.) [7], the C_20_ diterpene geranylgeranyldiphospate (GGPP) stands at the crux of a number of other biosynthetic pathways important to plant survival and adaptation. GGPP is also used as a substrate in the formation of dolichol (required for protein glycosylation) and phytol side chains during Chl biosynthesis. Changes in fluxes through many of these different pathways could influence other pathways through feedback regulation, changes to higher order regulatory genes and changes to the balance between substrate “draw”, which depends on tissue-specific and organ-specific gene expressions, and expression timing. The feedback regulation balances between substrate “draw” and expression timing are known for GA biosynthesis, the 2-C-methyl-derythritol-4-phosphate (MEP) pathway and for carotenoid biosynthesis [8], [9].

PQ transports electrons from PSII to cytochrome b_6_f in the light harvesting reactions of photosynthesis [1], [10] and is the immediate electron acceptor (co-factor) in the -carotene to lycopene stage during carotenoid and ABA biosynthesis and in the desaturation of phytoene and ζ-carotene (Figure 1). A certain quinone/hydroquinone balance is necessary for optimal phytoene desaturation. PQ is enriched in chloroplasts and is also found in Golgi membranes and in minor quantities in microsomes [11]. PQ is not detectable in mitochondria; instead the mobile quinone electron carrier, ubiquinone, dominates in mitochondria. The presence of PQ in the cytosol has also been reported, which probably reflects its transport from the site of synthesis to its final location [11], [12].

In higher plants, GAs can regulate seed germination, stem elongation, leaf expansion, trichome development and plant fertility (stamen elongation, pollen release and germination and pollen tube growth) [8], [13]–[15], GAs are mainly synthesized by the MEP/isoprenoid pathway and this biosynthesis can be divided into three stages [16]–[18]. The *GA1* and *GA2* genes play an important role in the conversion of GGPP to ent-kaurene and the Arabidopsis *ga1* mutant is a GA-responsive male-sterile dwarf due to the disruption of GA biosynthesis. Accumulation of the 1st stage enzyme, cyclase *ent*-copalyl diphosphate synthase (CPS, also known as GA1), in *Escherichia coli*, the conversion of GGPP to copalyl diphosphate (CDP) in GA1^+^/GGPP synthase^+^ transgenic *E. coli* and the transgenic complementation of Arabidopsis *ga1-3* mutants, confirmed that the *GA1* gene locus, U11034, encodes CPS [19]. The Arabidopsis *ga2* mutant is also a GA-deficient dwarf because it contains mutated ent-kaurene synthase (KS) and has impaired CDP conversion to ent-kaurene [20].

Genes encoding HST or its homologs have been isolated and identified in Arabidopsis and many other plants [2], [4], [21]–[23]. The HST gene, *VTE2*, was first isolated from *Glycine max* and the constitutive expression of *VTE2-2* in transgenic Arabidopsis resulted in a significant 13% tocopherol increase compared to the transgenic vector control plants [22]. Previous studies have explored the function of the Arabidopsis *HST* by utilizing *pds2* mutants and transgenic technology [2], [4], [21], [22]. Enzyme assay results for cell expression of the *HST* gene in *E. coli* suggested that HST catalyzes the first step in PQ biosynthesis [2]. Overexpression of the *HST* gene improved prenyl lipid, PQ and tocopherol levels in transgenic Arabidopsis [2], [22]. Disruption of the *HST* gene produced an albino phenotype and caused a deficiency in the synthesis of PQ and tocopherol by affecting the prenyl/phytyl transferase enzyme [4]. The in-frame 6 bp deletion in the coding region of the *HST* gene caused an albino phenotype and the expression of the *HST* gene in this *pds2* mutant turned albino plants into green plants [21].

Recently, we screened and found a new recessive albino knockdown mutant called *pds2-1*. TAIL-PCR and DNA sequencing showed that the mutant phenotype was caused by a T-DNA insertion in the *HST* gene. To date, phenotypic analysis of HST has been restricted to an albino phenotype, which results from disruption to chloroplast development and photosynthetic pigment biosynthesis. However, in addition to pigment biosynthesis, HST indirectly regulates carotenoid and ABA biosynthesis, but analyses of the developmental and the physiological changes due to *HST* gene blockage have not been reported. Detailed analysis of *pds2-1* indicated that *HST* knockdown impaired carotenoid and ABA biosynthesis, as well as GA biosynthesis and auxin content, which resulted in severe developmental defects. However, the application of exogenous GA_3_ and ABA to *pds2-1* partially restored the wild-type phenotype. Our new mutant line and analytical data confirm that HST is essential for carotenoid, ABA, and GA biosynthesis and that it plays a critical role in plant growth and development.

## Materials and Methods

### Plant Materials and Growth Conditions

*Arabidopsis thaliana* (ecotype Columbia-0) plants were grown in soil or on ½ strength Murashige and Skoog (MS) plates (pH 5.7) containing 0.8% agar and 3% sucrose at 22°C and 65% humidity under a 16 h light (100 µmol m^−2^ s^−1^)/8 h dark cycle. Insertion mutants were obtained from a transgenic experiment via the transformation of Arabidopsis by the floral dip method [24] using *Agrobacterium tumefaciens* GV3101 that contained pART27 [25]. Self pollinated seed recovered from 16 independent transgenic events was screened on ½ strength MS plates containing 50 mg L^−1^ of kanamycin. Then the surviving seedlings were transferred to soil to generate seeds at 22°C under long-day conditions. For PCR, RT-PCR, Enzyme-linked immunosorbent assays (ELISA), RNA-sequencing and microscope analyses, the seedlings were grown on ½MS plates containing 3% sucrose.

### TAIL-PCR

The CTAB method was used to isolate genomic DNA from WT seedlings and heterozygous Arabidopsis. Genomic DNA was used as a template for the TAIL-PCR, which was carried out using a Genome Walking Kit (TAKARA) according to the manufacturer's instructions. The three gene-specific primers used in TAIL-PCR were: R1 (5′-GTGCTGCAAGGCGATTAAGTTGGGTAA-3′), R2 (5′-ATTGCGTTGCGCTCACTGCCCGCTTTC-3′) and R3 (5′-GTGGCTCCTTCAATCGTTGCGGTTCTG-3′). The PCR products were sequenced using R3 as the sequencing primer. The sequencing results were searched against the Arabidopsis genome sequence database (GenBank) using BLAST to localize the position of the T-DNA insertion.

### Co-segregation Analysis

Three primers were designed and used to analyze the segregation pattern of the T-DNA insertion into the *HST* gene in seedlings that showed a mutant phenotype. The analysis was performed on DNA extracted from plates of WT and heterozygous mutant seedlings and the primer sequences were: hst-g1 (5′-CGAAAGTAAGCAGAGCAAAGAGT-3′), hst-g2 (5′-CATTCCCACAAATAAGAGCAAGA-3′) and R3 (5′-GTGGCTCCTTCAATCGTTGCGGTTCTG-3′). The PCR products were identified using 1% agar gel electrophoresis.

### Cloning of the *HST* Gene and Binary Vector Construction

Based on the *HST* cDNA (Accession No.: NM_001161137.1) sequence, a pair of primers: hst-1 (5′-AAACGAAAGTAAGCAGAGCAAAG-3′) and hst-2 (5′-CTAGAGGA- AGGGGAATAACAGATAC-3′), were designed to obtain the DNA sequence that included the coding region of the *HST* gene. Total RNA isolated from 3-week-old Arabidopsis was used for the reverse transcription-PCR. The PCR product was cloned into the T vector (TAKARA) and confirmed by DNA sequencing. The vector containing the *HST* full-length coding region was used as a template for binary vector construction, together with a pair of primers: HST-f (5′-cccgggccATGGAGCTCTCGATCTCACAATC-3′; where “cccggg” represents a *Sma* I restriction site and “ccATGG” represents a *Nco* I site) and HST-r (5′-tctagaactagtTTAGAGGAAGGGGAATAACAGATACT-3′; where “tctaga” represents a *Spe* I site and “actagt” represents a *Xba* I site). The PCR product was digested with *Nco* I and *Spe* I and cloned into the *Nco* I-*Spe* I site of the binary pCAMBIA1302 vector, creating 35S_pro_::*HST*, in which the *HST* gene was expressed from the *35S* promoter. The *HST* antisense plasmid, *Anti*-*HST*, was created by placing the *Xba* I-*Sma* I fragment of the PCR product between the 35S promoter and the NOS terminator of the binary pBI121 vector in an antisense orientation. The primers used for subcloning the *HST* gene promoter included Pro-HST-f (5′-atcgatGCAGACAATGTTAATGAAGAAGGCG-3′) and Pro-HST-r (5′-tctagaTTTGTGTCCAATCCTCTTTCCGG-3′). The PCR products were subcloned into the *Cla* I-*Xba* I site of pBI121, creating *HST*_pro_::*GUS*, in which the *GUS* gene is expressed driven by the *HST* promoter.

### *Agrobacterium* and Plant Transformation

The binary constructs described above were incorporated into *Agrobacterium tumefaciens* GV3101 using the freeze-thaw method [26]. Wild-type Arabidopsis was transformed with *Agrobacterium*, harboring Anti-*HST* or *HST*_pro_::*GUS*, using the floral dip method [24]. As homozygous *pds2-1* mutant plants could not generate seeds, heterozygous mutant plants (*PDS2-1*/*pds2-1*) growing in soil were transformed with *Agrobacterium* harboring 35S_pro_::*HST*. Transgenic plants were selected using 50 mg L^−1^ kanamycin (Anti-*HST* or HST_pro_::*GUS*) or 20 mg L^−1^ hygromycin B (35S_pro_::*HST*).

### RT-PCR and Quantitative Real-Time RT-PCR (qRT-PCR)

For RT-PCR, WT and albino Arabidopsis plant leaves were harvested after 3 weeks growth on agar medium containing 3% sucrose. Total RNA was extracted using Trizol Reagent (Biomed). The cDNA was synthesized with the reverse transcriptase (TaKaRa) and Oligo d(T) primer, and PCR was performed using the hst-1 and hst-2 primers. *UBQ10* (Access No.: NM_116771.5) was used as the inner control gene along with two primers: UBQ-f (5′-GCCGGAAAACAATTGGAGGATGGT-3′) and UBQ-r (5′-CATTAGAAAGAAAGAGATAACAGG-3′). RT-PCR images were captured using a Gel Image Analysis System.

qRT-PCR analysis was used for the expression analyses and was performed on 7-week-old WT plants at different developmental stages and on a variety of tissues. It was also performed on 7-week-old WT or *pds2-1* mutant plants for gene expression analyses. The system used was an ABI 7500 system with SYBR green detection. For analyses of *HST* expression in different tissues and developmental stages of WT Arabidopsis, *UBQ10* was used as an internal control. For analyses of gene expression in *pds2-1* mutants, *TUB2* was used as an internal control. The two-step thermal cycling profile used was 15 s at 95°C and 1 min at 68°C. The qRT-PCR reactions were performed in biological triplicates using total RNA samples extracted from three independent plant materials grown under identical growth conditions. The comparative ΔΔthreshold cycle method [27] was used to evaluate the relative quantities of each amplified product in the samples. All primers used in the qRT-PCR analyses are reported in Supplementary Table S1.

### RNA Sequencing

The whole plants of 3-week-old *pds2-1* and WT Arabidopsis were harvested and used for RNA extraction. The experiment was then repeated following the same collection scheme, thus providing two distinct biological replicates. Total RNA was extracted using TRIzol reagent following the manufacturer's instructions and was treated with RNase-free DNase I (NEB) to remove contaminated genomic DNA. The mRNA was isolated from the total RNA using Dynabeads oligo(dT) (Invitrogen). An illumina library was constructed using the NEB mRNA library prep kit instruction manual. The cDNA library was sequenced for paired ends on the Illumina Hiseq2000 platform at the Beijing Center for Physical and Chemical Analysis (Beijing, China). The raw reads were filtered by removing adaptor sequences, empty reads and low quality reads containing more than 50% bases with Q<30 using FASTX-Toolkit with methods described previously [28]–[30]. The resulting high-quality reads were mapped onto the *Arabidopsis thaliana* reference genome (TAIR 10) using Tophat (v2.0.5) [31]. Gene expression levels were measured as FPKM (fragments per kilobase of exon model per million mapped reads). EdgeR outputs the T-statistic and the p-values for each gene. Differential expression was estimated and tested using the edgeR software package (R version: 2.14, edgeR version: 2.3.52) [32]. We then calculated the FDR, estimated fold change (FC) and the FC log2 values. Transcripts that had an FDR≤0.05 and an estimated absolute log2 (FC)≥1 were determined to be significantly differentially expressed. Gene Ontology (GO), SEA and PAGE tools, found at the agriGO website (http://bioinfo.cau.edu.cn/agriGO/), were used to analyze the differential expression genes during the functional annotation and enrichment analysis.

### Chl and Carotenoid Assays

Chl and carotenoids were extracted from the aboveground parts of 40-day-old Arabidopsis, as described previously [33]. Absorbance (*A*) of the final solution at 663, 647 and 470 nm was measured and the concentrations of Chl a (Ca), Chl b (Cb) and carotenoids (Cc) were calculated as follows [34]:
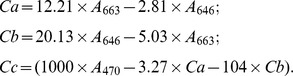



### ELISA Analyses

Initially, four plant hormones: ABA, GA_3_, IAA and ZR, were measured on the aerial parts of 3-week-old WT and *pds2-1* plants by a previously described ELISA method [35].

### β-Glucuronidase Staining

Histochemical β-Glucuronidase staining in *HST*_pro_::*GUS* transgenic plants was performed as described previously [36].

### Transmission Electron Microscope (TEM) and scanning electron microscope (SEM) Assays

Leaves harvested from 3-week-old WT and *pds2-1* plants were soaked in fixation buffer (4% glutaraldehyde in 100 mM cacodylate buffer, pH 7.2) and post-fixed for 6 h in secondary fixation buffer (1% OsO_4_ in 100 mM cacodylate buffer, pH 7.4) at 4°C. The fixed samples were dehydrated, embedded in Spurr resin and cut into ultrathin sections. The specimens were subsequently stained with uranyl acetate and observed with a TEM (Hitachi).

For SEM analysis, leaves of 3-week-old WT and *pds2-1* plants were harvested and the analysis was performed as described previously [37]. The specimens were examined by a SEM (Hitachi).

## Results

### Phenotype Analysis of the Albino Mutant

**Figure 2:**
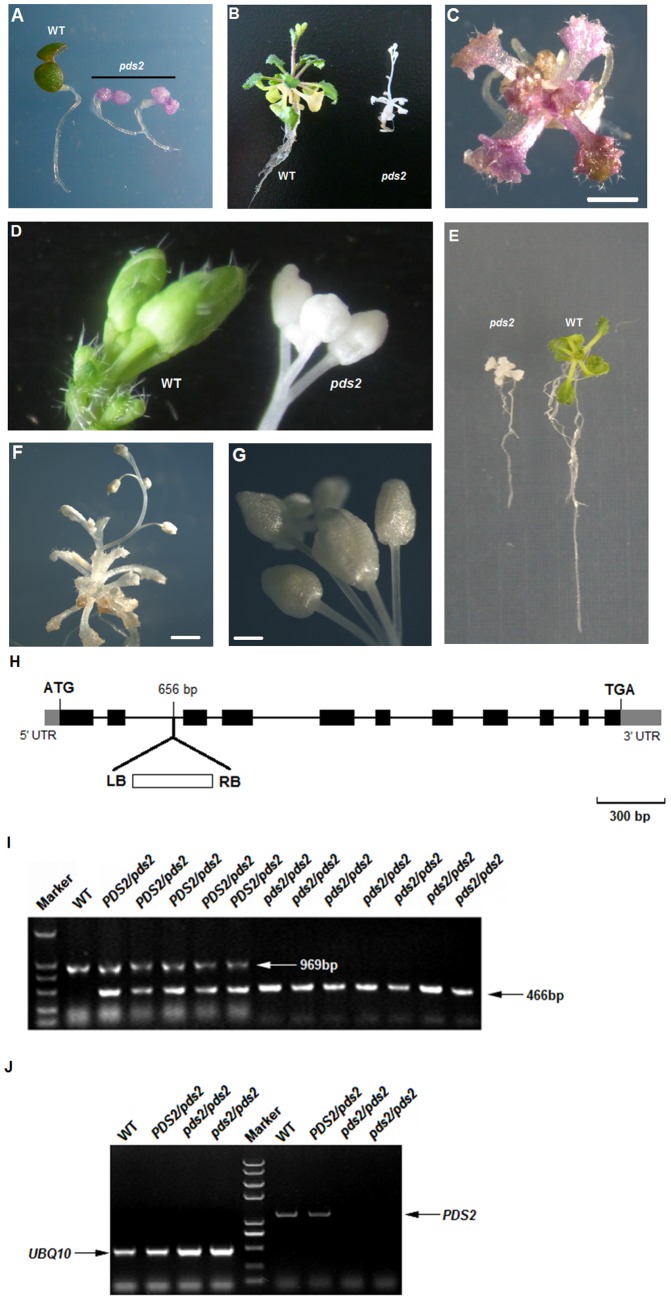
Analysis of *pds2-1* mutant plants. (**A**) WT and homozygous *pds2-1* (T_3_) seedlings with purple cotyledons grown on ½ strength MS medium for 4 days. (**B**) Adult homozygous *pds2-1* mutant (T_3_) and WT plants grown on ½-strength MS medium for 4 weeks. (**C**) A purple T_3_ *pds2-1* mutant seedling. Scale bar = 1 mm. (**D**) Inflorescences and flower buds from 8-week-old WT and *pds2-1* plants. (**E**) Roots from *pds2-1* and WT plants. (**F**) Adult *pds2-1* plant grown on ½ strength MS medium for 8 weeks. Scale bar = 2 mm. (**G**) Adult inflorescence of a *pds2-1* mutant plant. Scale bar = 0.5 mm. (**H**) *HST* gene structure and the T-DNA insertion site in *pds2-1* mutants. Black boxes: *HST* gene exons; black lines: *HST* gene introns. (**I**) Co-segregation analyses of the HST transgene with *pds2-1* mutant phenotypes. Marker: DNA marker; *PDS2/pds2*: heterozygous *pds2-1* mutants; *pds2/pds2*: homozygous *pds2-1* mutants. (**J**) RT-PCR analysis of the *HST* gene in WT and *pds2-1* mutant plants. The *UBQ10* gene was used as an internal control.

**Figure 3:**
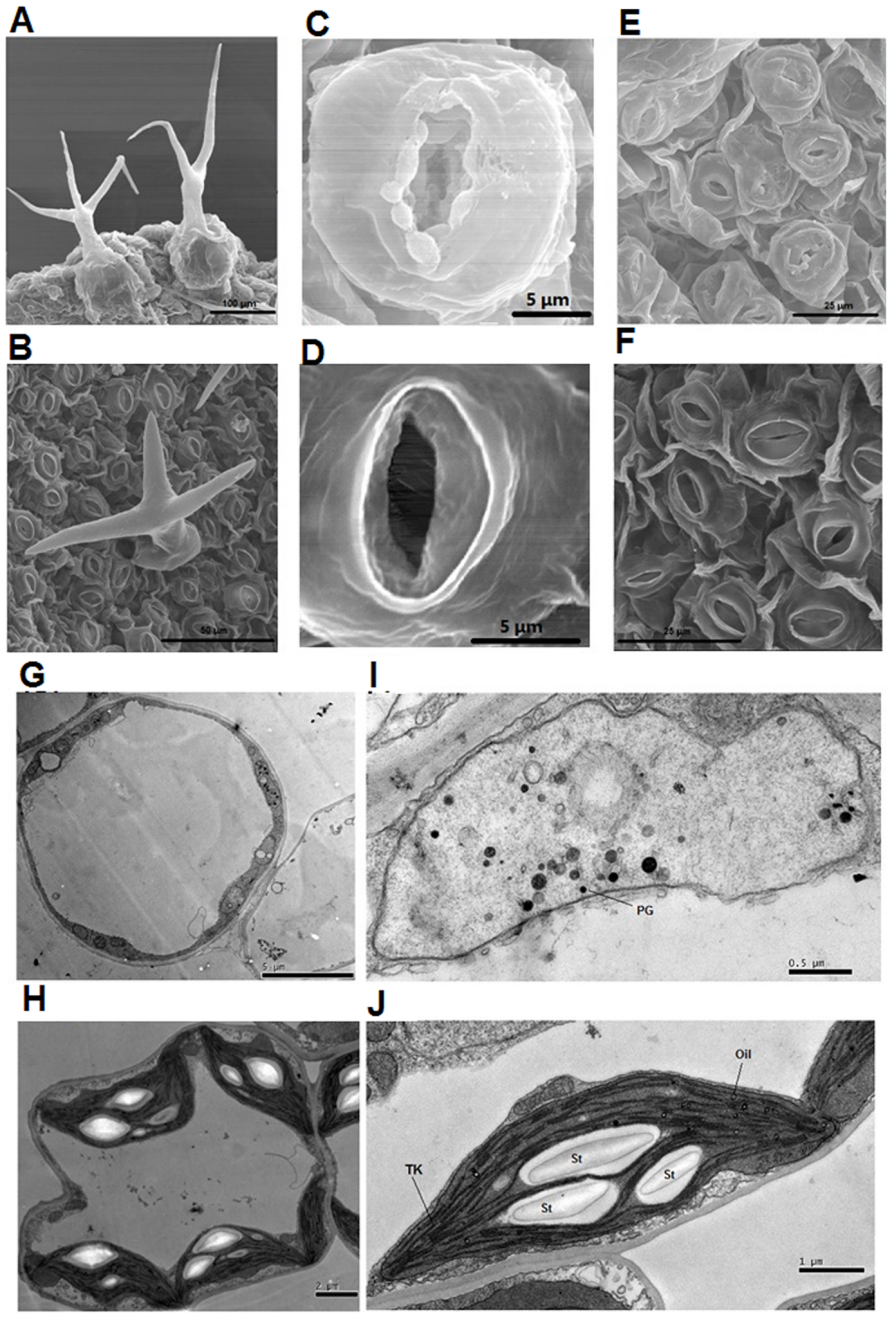
SEM and TEM of mesophyll cells and leaf surfaces of *pds2-1* mutant plants. (**A**) SEM analysis of trichomes from *pds2-1*. Scale bar = 100 µm. (**B**) SEM analysis of the trichomes from a WT plant. Scale bar = 50 µm. (**C,D**) SEM analysis of stomata from a *pds2-1* plant (**C**) and a WT plant (**D**). Scale bars = 5 µm. (**E,F**) SEM leaf surface analysis of a *pds2-1* plant (**E**) and a WT plant (**F**). Scale bars = 25 µm. (**G**) TEM analysis of a mesophyll cell from a *pds2-1* plant. Scale bar = 5 µm. (**H**) TEM analysis of a mesophyll cell from a WT plant. Scale bar = 2 µm. (**I**) TEM analysis of chloroplasts from a *pds2-1* plant. PG: plastoglobule. Scale bar = 0.5 µm. (**J**) TEM analysis of chloroplasts from a WT plant. St: starch granule; Tk: thylakoid; Oil: oil drops. Scale bar = 1 µm.

*Arabidopsis thaliana* was transformed by the binary vector, pART27, so that a small number (16) of T-DNA insertion lines could be generated. Seedlings from self-pollinated plants, selected on ½-strength Murashige and Skoog plates containing kanamycin and sucrose, were mainly green, but one plate contained several albino plants. Progeny from heterozygous albino plants were mainly green, but progeny derived from self-pollinated heterozygotes segregated 3∶1 into green and albino colored siliques (Figure S1). The cotyledons of all the young homozygous progeny from the albino type plants were purple on the selection medium (Figure 2 A), which suggested that Chl may be low or absent and that anthocyanin pigments may have accumulated. Most homozygous albino mutants gradually faded to white with continued growth on the selection medium. A small number of plants also showed the purple coloration on the rest of their plant parts (Figure 2 A–C). All albino seedlings died when they were grown on soil or ½ strength MS medium without sucrose, but in ½ MS medium with 3% sucrose, albino seedlings produced albino leaves (and even translucent stem tissues) and only one main shoot over their whole life cycle (Figure 2 B,E,F). Low resolution microscopy indicated that the albino plants had shorter roots, fewer root hairs, fewer and smaller leaves with shorter petioles and a reduced trichome density (Figure 2 B–F; Figure S2; Figure S3). Microscope or SEM was used to investigate the leaf surface morphology (trichomes and stomata) of *pds2-1* and WT plants in greater detail. The results showed that *pds2-1* trichomes were shorter in length and most (∼80%) trichomes had two branches instead of three (Figure 3 A,B; Figure S3; Figure S4). Their stomata had larger openings with unusual swollen structures surrounding the opening (Figure 3 C–F), which suggested that *pds2-1* stomata may close abnormally.

### Albino and Dwarf Phenotypes are Caused by a T-DNA Insertion into *HST*

We hypothesized that the albino *pds2-1* phenotype was caused by a T-DNA insertion because the albino plants were obtained from transgenic Arabidopsis T-DNA insertion lines and the green and albino plants of T_1_, T_2_ and T_3_ segregated in a 3∶1 ratio on nonselective medium. To determine the insertion site within the Arabidopsis genome, DNA was isolated from T_3_ heterozygous plants and non-transformed WT plants (as a negative control) for the TAIL-PCR (Figure S5). The TAIL-PCR and sequencing showed that the insertion was located at Chr3:3782298, which is within the second intron of the Arabidopsis *HST* gene (At3g11945) (Figure 2 H).

To check whether the T-DNA insertion was responsible for the albino phenotype, DNA was isolated from WT, heterozygous (green) and homozygous (white) Arabidopsis plants for PCR analysis. Primers, hst-g1 and hst-g2 (Table S1) amplified a full-length 969 bp fragment of *HST* gDNA from the WT and heterozygous plants. In contrast, primers R3 and hst-g2 amplified a 466 bp fragment from the heterozygous and homozygous *pds2-1* plants. These results indicated that the T-DNA insertion in the *HST* gene co-segregated with the albino phenotype (Figure 2J). To analyze the transcription of *HST* in the heterozygous and homozygous *pds2-1* plants, RT-PCR was conducted using gene specific primers: hst-1 and hst-2. The results showed that *HST* expression was eliminated in the homozygous plants (Figure 2 J), which suggested that albino plants were *HST* null mutants and that the mutant phenotype was caused by a T-DNA insertion into the *HST* gene.

### The *HST* Gene is Highly Expressed in Green Tissues

**Figure 4:**
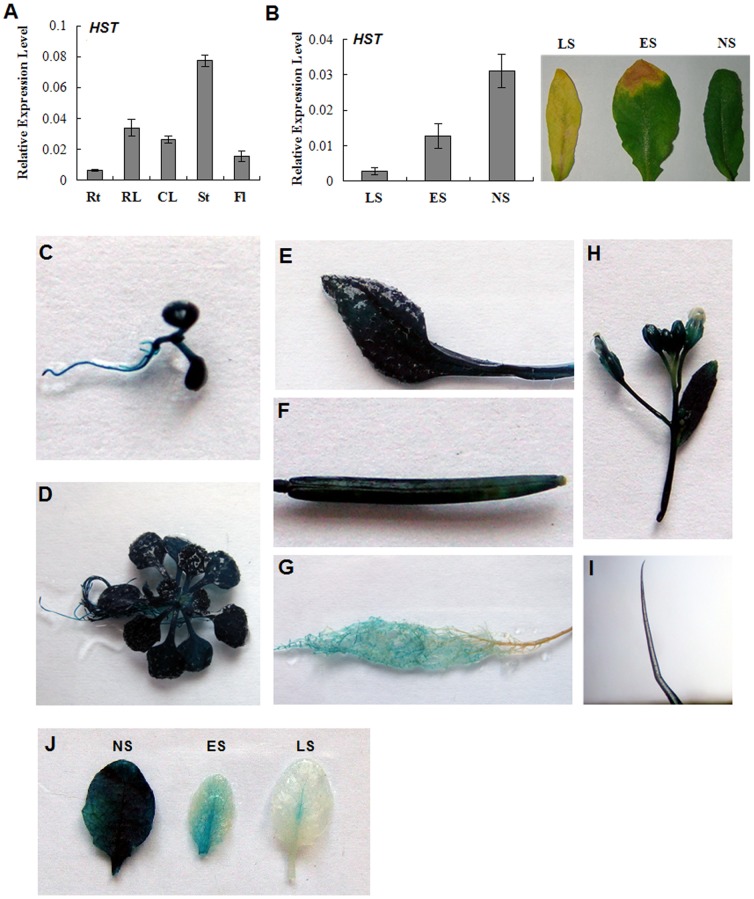
Expression analysis of the *HST* gene in WT Arabidopsis. (**A**) Tissue-specific expression as determined by qRT-PCR. The *UBQ10* gene was used as an internal control. Values are means ± SD (n = 3). Rt: roots; RL: rosette leaves; CL: cauline leaves; St: stems; Fl, flowers. (**B**) *HST* transcript levels at different leaf development stages. NS: no senescence; ES: early senescence; LS: late senescence. (**C–J**) Representative GUS expressions in HST_pro_::*GUS* transgenic Arabidopsis. (**C**) 5-day-old etiolated seedling; (**D**) 15-day-old seedling; (**E**) mature leaf of a 4-week-old plant; (**F**) mature silique; (**G**) root; (**H**) inflorescence and flowers; (**I**) trichome from a stem; (**J**) leaves at different developmental stages.

To understand the breadth of the role played by the *HST* gene, we re-examined the expression pattern of *HST* in WT Arabidopsis using qRT-PCR. The qRT-PCR assays revealed that *HST* was expressed at high levels in stems and leaves, but at relatively lower levels in the flowers and roots of adult plants (Figure 4 A). Furthermore, different *HST* expression levels were detected in the leaves at different developmental stages. The HST transcript levels were highest in non-senescent leaves, but they gradually decreased as the leaves began to senesce (Figure 4 B).

To further examine the *HST* expression patterns in different plant tissues, a 813 bp *HST* promoter sequence, upstream of the transcription start site, was fused to a GUS-coding sequence and this led to the appearance of HST_pro_::*GUS* transgenic Arabidopsis plants. In 40-day-old seedlings, GUS activity was highly detected in green tissues, such as leaves and stems (Figure 4 C–J). Notably, GUS activity was detected at a high level in adult green leaves, but at decreased levels in senescent leaves (Figure 4 J). These results suggested that *HST* was highly expressed in green tissues and had an important role to play in their development.

### *HST* is Required for Pigment Accumulation, Proplastid Growth and Thylakoid Membrane Formation

Total Chl and carotenoid (Car) contents were examined in *pds2-1* and WT plants. The calculated results showed that Chl and Car were almost absent in the homozygous *pds2-1* mutant plants. Chl a decreased to 0.55% of the WT level, Chl b to 1.86% and total Chl to 0.93% (Table 1), while carotenoids were reduced to 0.24% of the WT level. The Chl a/b ratio also decreased dramatically in *pds2-1* mutants and was only 26.90% of the WT level. As a result, the Chl/Car ratio increased to 20.79 in the *pds2-1* mutants (Table 1). These results showed that disruption of the *HST* gene caused significant reductions in Chl and carotenoid levels and that carotenoids were affected more seriously than Chl in *pds2-1* mutants.

**Table 1:** Chl and carotenoids contents of WT and *pds2-1* mutant plants.

Arabidopsis	Chl a (mg L^−1^)	Chl b (mg L^−1^)	Car (mg L^−1^)	Chl (mg L^−1^)	Chl a/b	Chl/Car
WT	19.03±4.60	8.01±1.86	5.17±0.82	27.04±2.76	2.60±0.93	5.29±0.38
*pds2-1*	0.10±0.01**	0.15±0.01**	0.01±0.00**	0.25±0.01**	0.70±0.03**	20.79±3.25**

Leaves from 40-day-old plants were sampled to determine the Chl and carotenoid contents.

** indicates significant differences of the means at P<0.01 between WT plants and *pds2-1* mutant plants for each parameter measured (n = 4).

To assess the effect of *pds2-1* mutations on chloroplast development, plastids from the first leaves of 30-day-old seedlings were examined by TEM. The data showed that the chloroplasts in *pds2-1* were smaller than those in the wild type and lacked starch granules, oil drops and thylakoids, but contained many densely stained globule structures that resembled plastoglobules (Figure 3 G–J). These results suggested that proplastids in the mesophyll cells of the *pds2-1* mutant have lost the ability to develop into mature chloroplasts. The RuBisCO large subunit levels were also highly reduced in *pds2-1* when total protein was analyzed by SDS-PAGE (Figure S6) and this was consistent with the albino phenotype and the chloroplast development defects in *pds2-1*. These data suggested that *HST* played an important role in pro-plastid growth and thylakoid membrane formation.

An anti-sense HST-RNAi vector was used to transform Arabidopsis WT plants and six transgenic Arabidopsis lines were analyzed for phenotype and chloroplast defects (Figure S7 A). Leaves from one adult RNAi transgenic line (Anti-HST) were a lighter green color than the WT plant leaves. The *HST* transcripts were significantly down-regulated in this line compared to the WT plants and the darker green RNAi lines (Figure S7 B,C). The levels of Chl a and b were also significantly lower in Anti-HST plants compared to the WT lines (Figure S7 D).

### The *HST* Gene Rescues the *pds2-1* Mutant Phenotype

**Figure 5:**
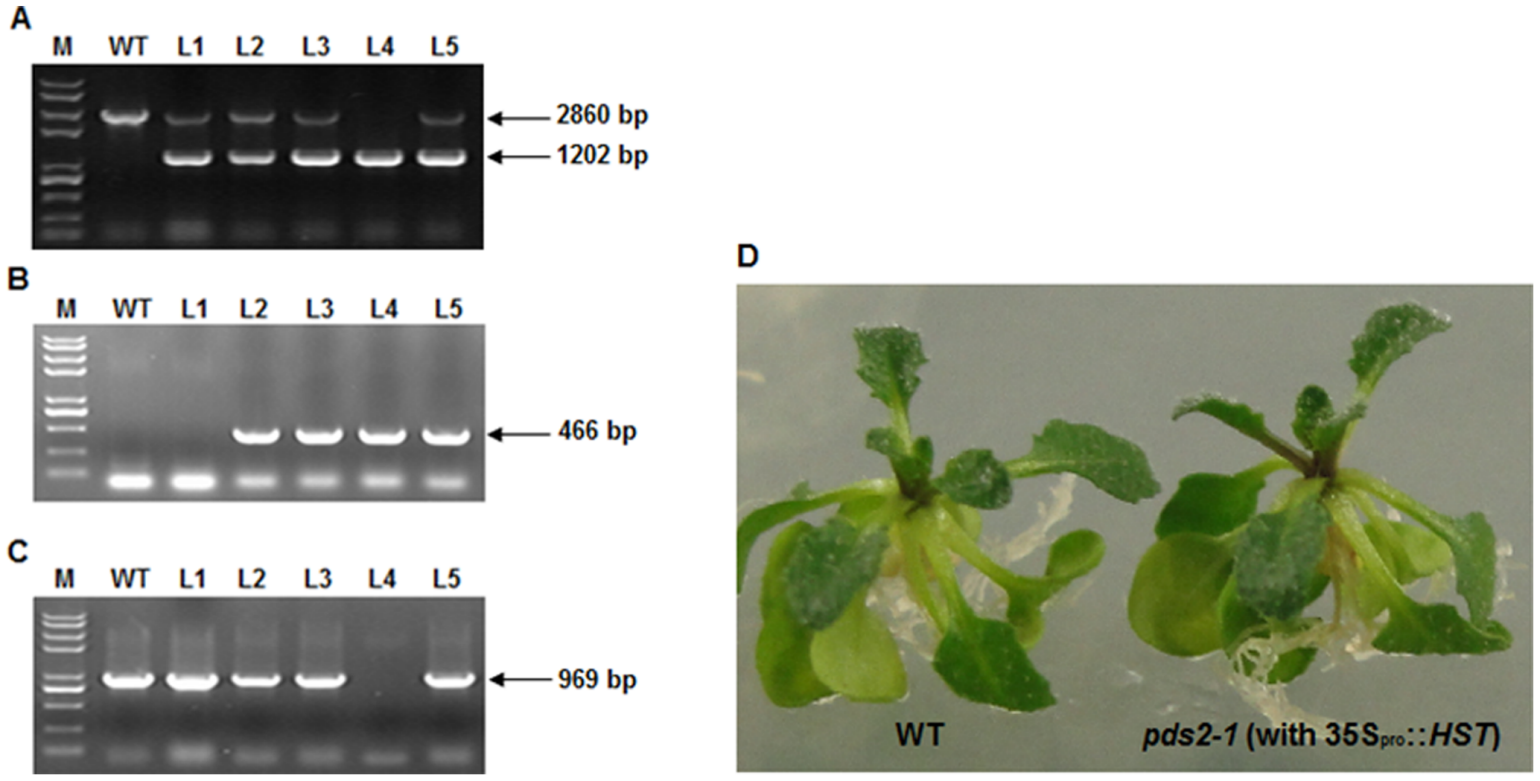
*HST* gene rescues the phenotype of *pds2-1* mutant plants. (**A**) PCR analysis of transgenic Arabidopsis using the HST-f and HST-r primers. The 2,860 bp band represents *HST* gDNA and the 1,202 bp band represents the HST coding domain sequence in transgenic Arabidopsis. M: DNA marker; WT: wild type; L1-5: five independent plants transformed with 35S_pro_::*HST*. (**B**) PCR analysis of transgenic Arabidopsis using the R3 and hst-g2 primers. The 466 bp band represents the T-DNA insertion in the *HST* gene. (**C**) PCR analysis of transgenic Arabidopsis using the hst-g1 and hst-g2 primers. Absence of the 969 bp band shows that L4 is a *pds2-1* homozygous mutant. (**D**) Representative phenotype of *pds2-1* homozygous plants rescued by transforming them with 35S_pro_::*HST*.

*HST* cDNA, driven by the 35S promoter, was inserted into the genome of the *pds2-1* heterozygous mutant. Transgenic seeds were screened on ½MS plates containing 3% sucrose and 20 mg L^−1^ hygromycin and six independent lines were selected. Homozygous *pds2-1* plants with *35S_pro_*::*HST* were confirmed by PCR (Figure 5 A–C). T_2_ progeny of *pds2-1* that were homozygous for *35S_pro_::HST* displayed a phenotype that was similar to the WT plants (Figure 5 D). These results showed that the *HST* gene driven by the 35S promoter could completely rescue the mutant phenotype.

### Severe Declines in GA and ABA Content Were Seen in *pds2-1* Leaves

ELISA were performed to determine the concentrations of ABA, GA_3_, zeatin riboside (ZR) and IAA in the aerial parts of 3-week-old WT and *pds2-1* plants. A substantial decrease in ABA (43.1%), GA_3_ (61.5%) and ZR (69.8%) concentrations occurred in *pds2-1* plants compared to WT plants (Table 2) but the ratio of GA_3_:ABA was increased from 0.0621 in WT to 0.0923 in *pds2-1* (Table 2). This disruption of the *HST* gene also led to a substantial increase (by 41.6%) in the IAA content of *pds2-1*.

**Table 2:** Plant hormone concentrations in *pds2-1* and WT seedlings grown on ½ MS plates.

Plant hormone	WT (ng·g^−1^·FW)	*pds2-1* (ng·g^−1^·FW)	*pds2-1*:WT	P-value
ABA	138.38±2.95	59.58±2.68	0.431	<0.0001
GA_3_	8.95±0.24	5.5±0.15	0.615	<0.0001
ZR	15.94±0.56	11.12±0.43	0.698	<0.0001
IAA	56.49±2.14	79.98±2.18	1.416	0.0002

Three-week-old seedlings were sampled to determine the levels of four plant hormones. P-values represent significant differences of the means between WT and *pds2-1* tissues (n = 3) after comparing them using Student's t-test. FW: fresh weight.

### Gene Transcriptions That Specified Carotenoids, GA, ABA, Trichomes and Roots Were Depressed in *pds2-1*

**Figure 6:**
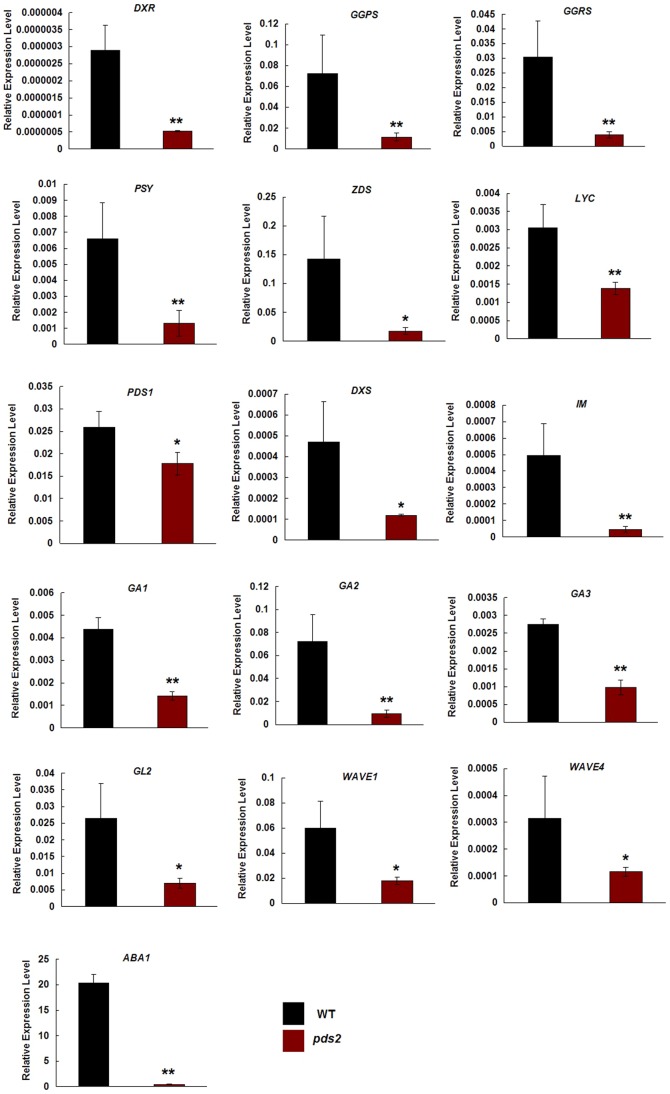
qRT-PCR analyses of genes involved in the carotenoid, GA and ABA biosynthetic pathways and in the regulation of trichome and root hair development. Significant differences of the means (± SD) between WT plants and *pds2-1* plants (n = 3) are indicated by * (P<0.05) and ** (P<0.01). The Arabidopsis *TUB2* gene was used as an internal control.

Previous reports indicated that albino mutants blocked the MEP pathway and led to a decrease in carotenoid, GA and ABA biosynthesis [33]. Therefore, we investigated whether disruption of the *HST* gene changed the expression of genes involved in these pathways using qRT-PCR. *DXS*, *DXR*, *LYC*, *IM*, *PSY* and *ZDS* are involved in the MEP pathway and in the biosynthesis of carotenoids [38]–[40]. GGPS encodes an enzyme involved in isoprenoid biosynthesis [41] and GGRS encodes a protein with geranylgeranyl reductase activity [42]. The *PDS1* gene (encoding the p-hydroxyphenylpyruvate) plays an important role in the synthesis of both plastoquinone and tocopherols in plants [43], *GA1*, *GA2* and *GA3* are involved in GA biosynthesis [19], [20], [44]; *ABA1* plays an important role in ABA accumulation [45] and *GL2*, *WAVE1* and *WAVE2* are involved in trichome and root hair initiation [46]. The results showed that transcription of all these selected genes was significantly lower in *pds2-1* plants compared to WT plants and the key gene in ABA biosynthesis, *ABA1*, was almost absent in the *pds2-1* plants (Figure 6).

### A Mutation in *HST* Promotes Early Flowering in *pds2-1*

**Figure 7:**
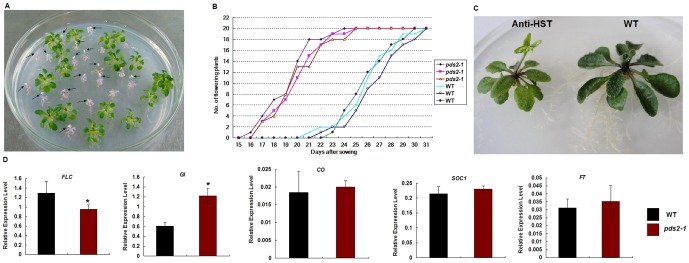
Reduced expression of *HST* results in an early flowering phenotype. (**A**) Typical 22-day-old *pds2-1* and WT seedlings. Black arrows indicate floral structures on bolting *pds2-1* plants. (**B**) Onset of early flowering in three *pds2-1* plants compared with WT plants. (**C**) Anti-HST early flowering phenotype. (**D**) Expression analysis by qRT-PCR of genes involved in regulating flowering time in *pds2-1* plants. Significant differences of the means (± SD) between WT plants and *pds2-1* plants (n = 3) are indicated by * (P≤0.05). The Arabidopsis *TUB2* gene was used as an internal control. *FLC*: *Flowering Locus C*; *GI*: *Gigantea*; *CO*: *Constans*; *SOC1*: *Supressor of Overexpression of Constance1*.

Although the growth of homozygous mutants was severely retarded, the *pds2-1* albino plants could bolt when grown on ½MS containing 3% sucrose (Figure 7 A). All the homozygous plants had flower-like structures that never matured into normal flowers (Figure 7 A,B) and the flowers and stems were usually glabrous (Figure 2 D). Therefore, *psd2-1* had to be propagated in the heterozygous state, which suggested that the gene was essential for plant development. Moreover, flowering time was disrupted in *pds2-1* plants and they flowered earlier than the WT plants (Figure 7 A,C). This conclusion is supported by the light green-colored anti-sense HST-RNAi line, anti-L2, which also displayed an early flowering phenotype (Figure 7 D). Transcripts for two flowering genes, *FLC* and *GI*, were also significantly depressed in *pds2-1* plants, but *CO*, *SOC1* and *FT* were unaffected by the mutation (Figure 7 E).

### *HST* Disruption Affects the Expression of Many Photosynthetic Genes, Cellular Components and Biological Processes

**Figure 8:**
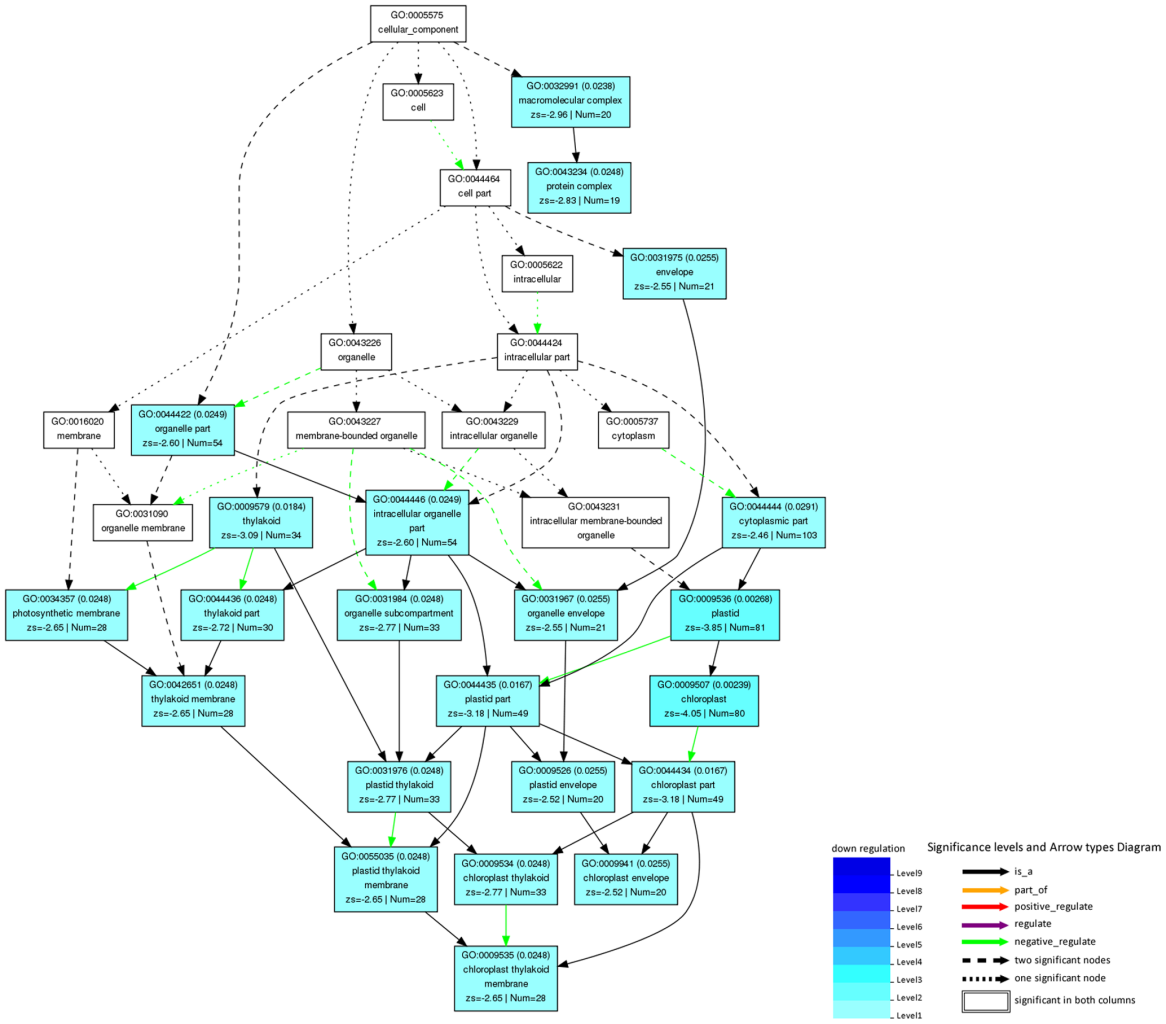
Hierarchical tree of over-represented GO terms (subcellular structure category) generated by parametric analysis of gene set enrichment. Boxes in the graph represent GO terms as labeled by their GO ID, term definition and statistical information. Significantly over-represented terms (adjusted P<0.05) are marked with color, while non-significant terms are shown as white boxes. A box's degree of color saturation is correlated to the representation level of the term. Boxes connected by dotted, dashed and solid lines represent zero, one and two down-regulation terms at the ends connected by the line, respectively.

RNA sequencing was carried out to further explore the effects of the *HST* gene on metabolic pathways. A total of 1254 unique genes exhibited two fold or greater differential expression in *pds2-1* plants compared to WT plants, with 351 genes up-regulated and 903 genes down-regulated. A total of 222 differentially expressed genes were then identified with a false discovery rate (FDR) of less than 0.05 (Table S2). Gene Ontology (GO) was used to analyze the differentially expressed genes by functional annotation and enrichment analysis, which required the use of singular enrichment analysis (SEA) and the Parametric Analysis of Gene set Enrichment tool (PAGE) [47]. In total, 212 of the 222 differentially expressed genes were assigned to at least one term in GO under the different biological process categories (molecular function, subcellular structure/components and biological process) and the number of significant GO terms were 138. In *pds2-1* mutants, the expression levels of many genes related to cellular structure/components, including thylakoids, plastids, chloroplasts, photosynthetic membrane, plastoquinone, plastoglobules, light-harvesting complexes, organelles, the cytoplasm and the apoplast, were dramatically reduced (Table S3). Moreover, the expression levels of molecular components involved in photosynthesis, the light reaction, pigment metabolic processes and processes such as the regulation of catalytic activity, response to stimuli, and response to stress, had also decreased significantly. When differentially expressed genes involved in cellular components were submitted to the PAGE tool after RNA sequencing, 22 GO terms (including thylakoid, chloroplast, photosynthetic membrane, etc.) were significantly over-represented in the hierarchical tree (Figure 8; Table S4).

### GA_3_ and ABA partially rescued the *pds2-1* mutant phenotype

**Figure 9:**
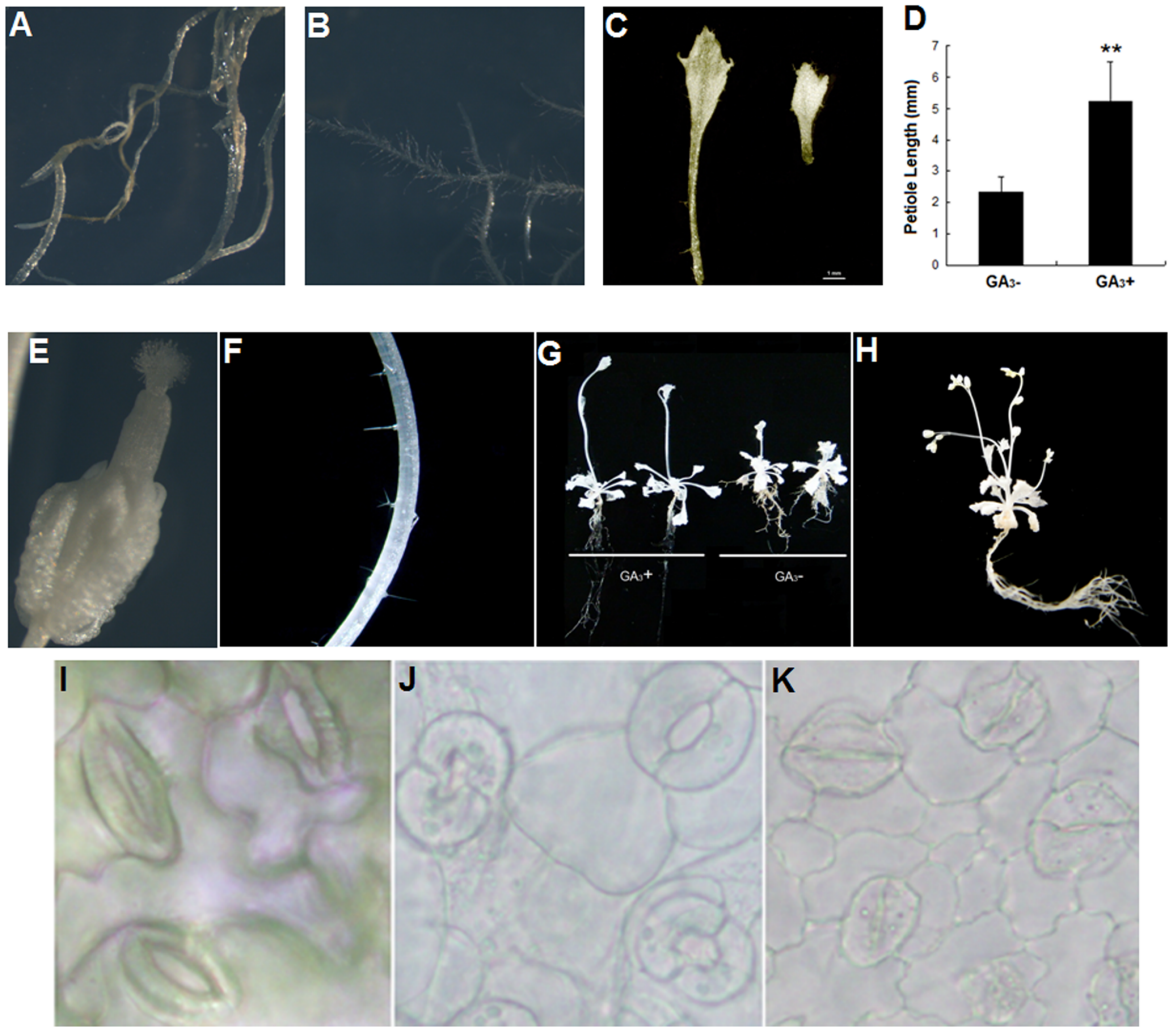
Exogenous application of GA_3_ or ABA to *pds2-1* mutants. (**A**) Root hair of a *pds2-1* mutant plant after 10 µM GA_3_ treatment. Scale bar = 1 mm. (**B**) Root hair of a *pds2-1* mutant plant without GA_3_ treatment. Scale bar = 1 mm. (**C**) Representative petiole lengths from *pds2-1* mutants with exogenously applied GA_3_ (GA_3_+) or without GA_3_ (GA_3_−). Scale bar = 1 mm. (**D**) Petiole length analyses of *pds2-1* plants with or without GA_3_ treatment. A total of 30 leaves (from 10 plants) were measured for each sample. ** indicates significantly different means (± SD) at P<0.01 compared to those without GA_3_ treatment. (**E**) The longer flower bud pistil seen on *pds2-1* mutant plants after GA_3_ treatment. Scale bar = 0.5 mm. (**F**) Trichomes (arrows) on a *pds2-1* mutant stem after GA_3_ treatment. (**G**) Sizes of bolting *pds2-1* mutant plants grown on ½ strength MS medium with or without GA_3_ treatment. (**H**) Mature *pds2-1* mutant plant showing multiple flowing shoots after GA_3_ treatment. (**I**) Usual stomata structure of a WT plant without ABA treatment. Scale bar = 5 µm. (**J)** Swollen open stomata structure of a *pds2-1* mutant without ABA treatment. Scale bar = 5 µm. (**K**) Closed stomata structure of a *pds2-1* mutant after 10 µM ABA treatment. Scale bar = 5 µm.

The 14-day-old *pds2-1* seedlings were transferred onto ½MS medium containing 3% sucrose and 10 µM GA_3_. Two weeks later, the leaf petioles and roots of all the tested *pds2-1* plants were longer than those grown without GA_3_ and root hair density had increased (Figure 9 A–D, G). The pistils of *pds2-1* mutants grew longer with GA_3_ supplementation, but flower buds did not develop into mature flowers (Figure 9 E). Stem trichomes were obvious on *pds2-1* mutant plants when treated with GA_3_ (Figure 9 F). The *pds2-1* plants treated with GA_3_ also had extended petioles, were larger and grew faster than those without GA_3_ and they produced more branches and inflorescences (Figure 9 G,H).

ABA plays an important role in regulating stomata closure in plants. To find out whether the stomata structural defect in *pds2-1* plants was caused by ABA absence, 14-day-old *pds2-1* seedlings were transferred onto ½ MS medium supplemented with ABA. Two days later, the leaves of the mutant plants were harvested and the stomata structure observed using light microscopy. The stomata closed normally in *pds2-1* mutants with exogenous ABA (Figure 9 J–K), which suggested that the stomata closure defect was caused by an ABA deficiency in *pds2-1* mutants.

## Discussion

In this study, we identified an albino mutant, *pds2-1*, and showed that disruption to the *HST* gene resulted in an albino phenotype and developmental defects. Homozygous *pds2-1* mutants all died when they were grown on soil or ½MS medium without sucrose, but produced albino leaves or white inflorescences when 3% sucrose was included. Other Arabidopsis mutants exhibited a similar albino phenotype when DXR, PDS or the IspH homologue of an isoprene biosynthetic gene were disrupted [9], [33], [48], [49].

RT-PCR and GUS staining showed that *HST* was expressed in almost all the plant tissues and at higher levels in green tissues. HST protein contains a putative 69 aa chloroplast transit peptide at its N-terminus. Subcellular localization analysis of HST in a previous report showed that HST was targeted at chloroplasts [21]. In this study, TEM analysis demonstrated that *pds2-1* chloroplasts were smaller and developmentally malformed. Our study confirmed previous reports, which showed that *HST* disruption resulted in an albino phenotype in Arabidopsis [4], [21]. We measured Chl levels to confirm the negative effect of a mutated *HST* gene on the photosystem and found that the *pds2-1* mutant had significantly reduced Chl levels, which was consistent with the reduced expression we measured for key genes involved in chloroplast biosynthesis. Moreover, SDS-PAGE analysis of the total proteins in *pds2-1* plants also confirmed that the photosystem was destroyed and that RuBisCo was not produced after *HST* disruption. Transgenic mutants transformed with 35S_pro_::*HST* restored the normal green phenotype, but RNAi lines had yellowish-green leaves. Together, these results confirmed that *HST* was essential for normal plant growth and chloroplast development.

Carotenoids play important roles in many physiological processes and in chloroplast biogenesis. They are also precursors of ABA. Biochemical analysis of the *pds2-1* mutant plants revealed that carotenoids, ABA and PQ levels were dramatically reduced in plant leaves. HST is a critical enzyme in the PQ biosynthesis pathway. PQ is both a PSII electron carrier and a critical component that links carotenoid and ABA biosynthesis [4]–[6] with PS I, PS II and ATP synthesis [50]. Hence, the absence of PQ in *pds2-1* caused disruption to electron transport, which may have contributed indirectly to the developmental defects in chloroplasts via negative feedback during phytoene desaturation, carotenoid synthesis and chloroplast biogenesis. Inhibition of PQ biosynthesis, leading to phytoene accumulation, has also been reported in previously identified *pds2* mutants [4]. Our *pds2-1* mutant plants also displayed stomata closure defects and an application of exogenous ABA restored the wild type plant stomata. These results suggested that HST also had an indirect impact on stomata closure by affecting carotenoid and ABA biosynthesis through the regulation of PQ levels.

The *pds2-1* mutants showed a pronounced dwarf phenotype and trichome and root hair development defects. qRT-PCR analyses showed that three positive regulators (*GL2*, *WAVE1* and *WAVE4*) of trichome or root hair development were down-regulated in *pds2-1* mutants. ELISA analyses and qRT-PCR revealed that GA levels and the expression of GA biosynthesis genes, such as *GA1*, *GA2* and *GA3*, declined dramatically in *pds2-1* mutants. When exogenous of GA_3_ was added to the ½MS plates, the *pds2-1* plants grew larger and had more root hairs and branches. GAs play important roles in plant height, trichome initiation and root development [51]–[56]. In plastids, GGPP is a precursor of carotenoids, ABA, tocopherols, the phytol tail of Chl, plastoquinones and GA. Qin et al. (2007) showed that disruption of the phytoene desaturase gene, *PDS3*, in carotenoid biosynthesis impeded the biosynthesis of GAs [9] and a negative feedback mechanism controlled upstream genes in the *pds3* carotenoid mutant, which resulted in a decrease in GAs. Our novel GA findings, together with supporting literature and the other information, strongly suggest that HST disruption in *pds2-1* affects many pathways through direct and indirect mechanisms.

GAs play an important role in the positive regulation of flowering time [57], [58] and reduced gibberellin synthesis usually promotes late flowering phenotypes in Arabidopsis [59], [60]. Hence, *pds2-1* mutants should, in principle, exhibit late flowering because of the decrease in GA_3_. Instead, an unusually early flowering phenotype occurred in *pds2-1* and in the *anti-HST* plant. This early flowering phenotype was consistent with the decrease in the expression of a key flowering repressor, *FLC*, and an increase in the expression of the flowering regulator, *GI*, in *pds2-1* mutants. One interpretation of this data arises from the fact that ABA and GA levels decreased in *pds2-1* mutants. ABA is believed to act as an antagonist to GAs during plant growth and development [61], [62]. ABA biosynthesis mutants have an early flowering phenotype [61], [63] and ABA can delay flowering onset by up-regulating the expression of *FLC*, as occurs in Arabidopsis [64]. In fact, although the levels of both hormones fell in *pds2-1* seedlings, the overall ratio of GAs∶ABA increased by 48.6% (Table 2). Genetic analyses of the interaction between GAs and ABA shows that GAs play a major rate-limiting role in floral promotion [61]. Our findings, together with an earlier report, suggested that the increased ratio of GA∶ABA in *pds2-1* mutants had maintained the predominant role played by GA and caused the triggering of the early transition from vegetative to reproductive growth. A second interpretation is that *HST* disruption affected other hormone levels. Earlier reports showed that a number of hormones, including GAs, ABA and auxins, played important roles in regulating flowering time [65]. For example, exogenous IAA was able to induce flowering in long day plants [66]. Our ELISA results showed that auxin levels (IAA) increased by 41.6% in the *pds2-1* mutant compared to the WT plants. Hence, the early flowering phenotype displayed by *pds2-1* mutant plants could also be due to their increased IAA levels. In summary, disruption of the *HST* gene promoted flowering by affecting the levels of several plant hormones.

Screening of a small Arabidopsis T-DNA population uncovered a novel albino mutant *pds2-1* with a T-DNA insertion in the *HST* gene. This gene is known to play an important role in PQ biosynthesis. The *pds2-1* mutation resulted in a typical carotenoid-deficient phenotype, with reduced PQ, abnormal chloroplast development, reduced photo-protection and reduced PS II activity. These findings confirmed that the *HST* gene plays an important role in pro-plastid growth and thylakoid membrane formation. However, the mutation also produced novel IAA-enhanced, ABA-deficient and GA-deficient phenotypes that had not been reported previously. Furthermore, GA_3_ and ABA application partially rescued the mutant phenotype. Other novel phenotypes not previously reported for this gene include short roots and petioles, fewer leaf numbers, root hairs and trichomes, more swollen stomata, an early flowering date and high expression of *HST* gene in green tissues. Moreover, qRT-PCR and RNA sequencing confirmed that the mutant had a blocked MEP pathway and the genes that played a role in GA biosynthesis did not function. This detailed analysis of the *pds2-1* mutant paves the way for a comprehensive study of the physiological regulation of carotenoids, chloroplast components and other physiological processes by major plant hormones through the use of a combination of genetic, biochemical and molecular approaches. HST will probably lie at the heart of these processes as it appears to impact directly and indirectly on so many hormones and biological processes.

## Supporting Information

Figure S1 **Seed segregation in a silique from a heterozygous** ***pds2-1*** **mutant plant.**
(TIF)

Figure S2 **Microscopic analysis of root hair from the** ***pds2-1*** **mutant and WT Arabidopsis.**
(TIF)

Figure S3 **Trichome phenotype of leaves from** ***pds2-1*** **and WT Arabidopsis.**
(TIF)

Figure S4 **Microscopic and statistical analyses of stomata from the** ***pds2-1*** **mutant and WT Arabidopsis.**
(TIF)

Figure S5 **Representative TAIL-PCR analysis of heterozygous (** ***PDS2*** **/** ***pds2*** **) plants.**
(TIF)

Figure S6 **SDS-polyacrylamide gel electrophoresis showing loss of a major protein subunit (RuBisCo) in** ***pds2-1*** **.**
(TIF)

Figure S7 **Gene expression and pigments in** ***HST*** **RNAi transgenic lines.**
(TIF)

Table S1 **Primers used in RT-qPCT analyses.**
(DOC)

Table S2 **Significant differential expression genes in WT and** ***pds2-1*** **.**
(DOC)

Table S3 **GO terms in all three categories (Biological Process, Cellular Component and Mollecular Function) generated by SEA.**
(DOC)

Table S4 **GO terms in Sub-Cellular Structure/Components generated by PAGE.**
(DOC)
