# Supplementary figures and images for: Disruption of the Homogentisate Solanesyltransferase Gene Results in Albino and Dwarf Phenotypes and Root, Trichome and Stomata Defects in Arabidopsis thaliana
Source: PLoS One. 2014 Apr 17;9(4):e94031. doi: 10.1371/journal.pone.0094031 (PMC3990575; doi:10.1371/journal.pone.0094031)

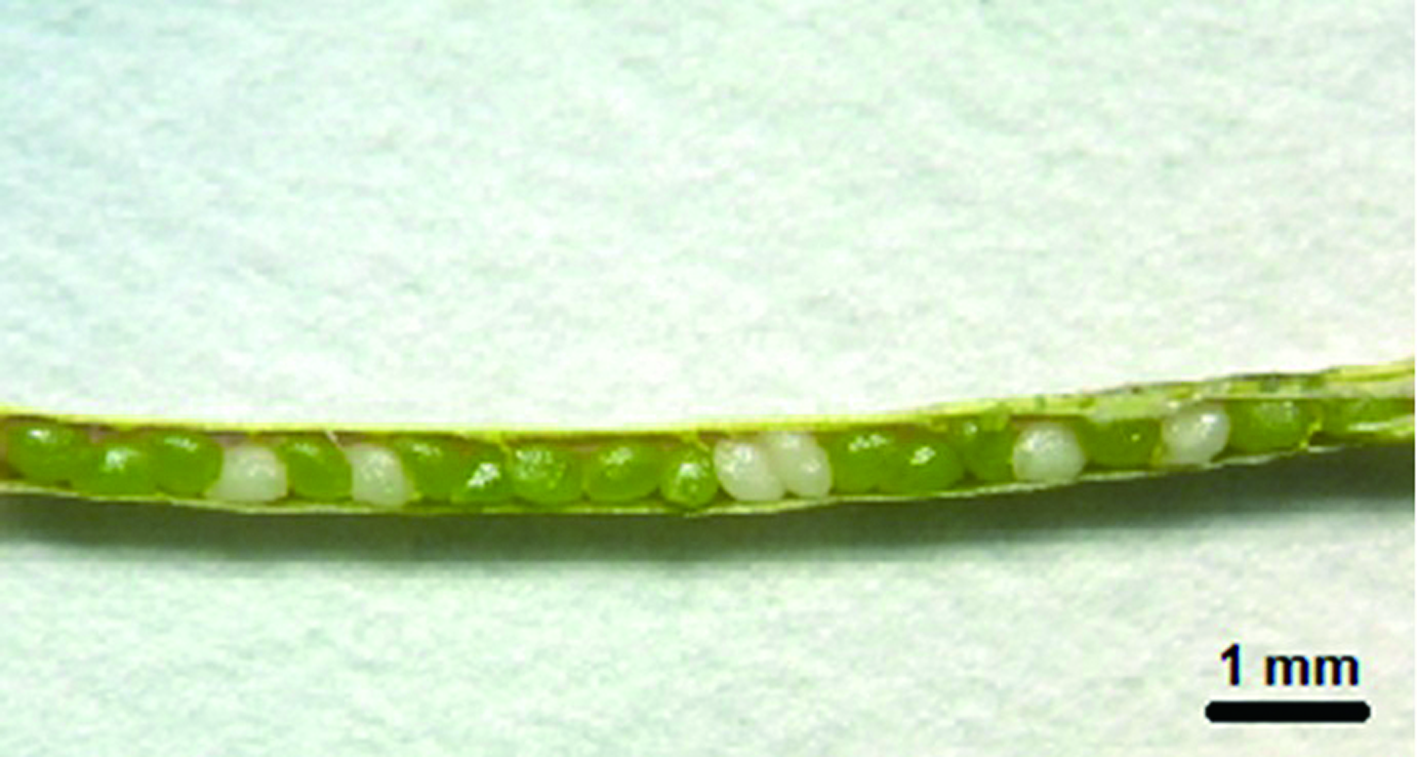

Supplement: Figure S1 — Seed segregation in a silique from a heterozygous pds2-1 mutant plant. (TIF) [file pone.0094031.s001.tif]

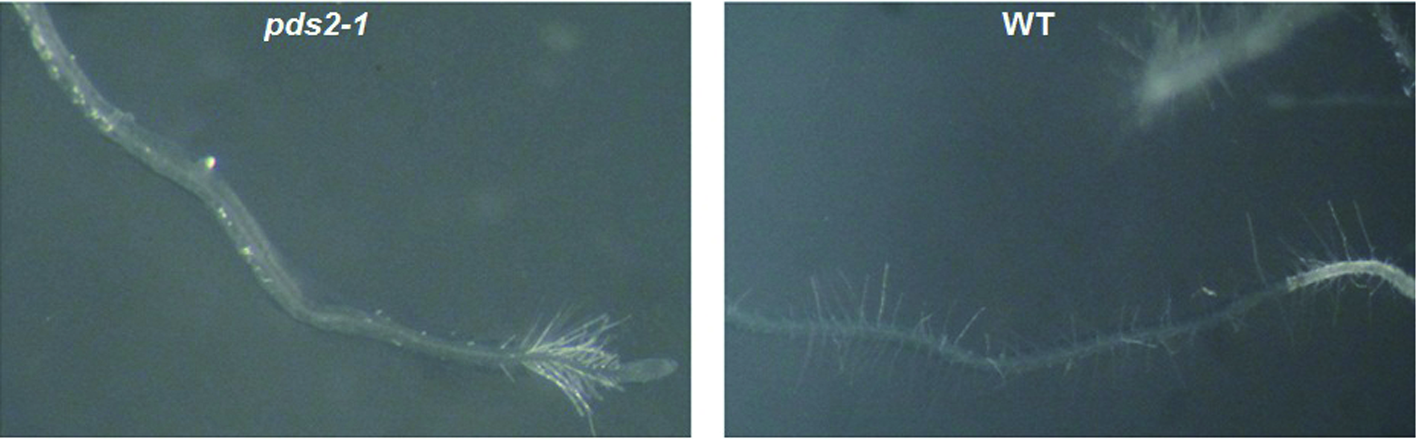

Supplement: Figure S2 — Microscopic analysis of root hair from the pds2-1 mutant and WT Arabidopsis. (TIF) [file pone.0094031.s002.tif]

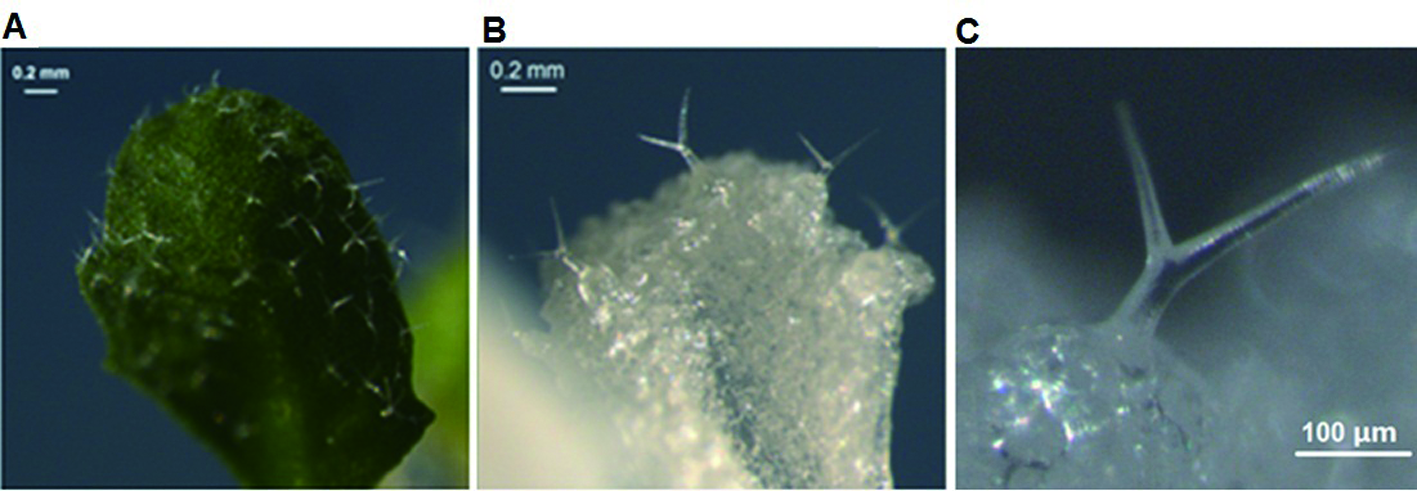

Supplement: Figure S3 — Trichome phenotype of leaves from pds2-1 and WT Arabidopsis. (TIF) [file pone.0094031.s003.tif]

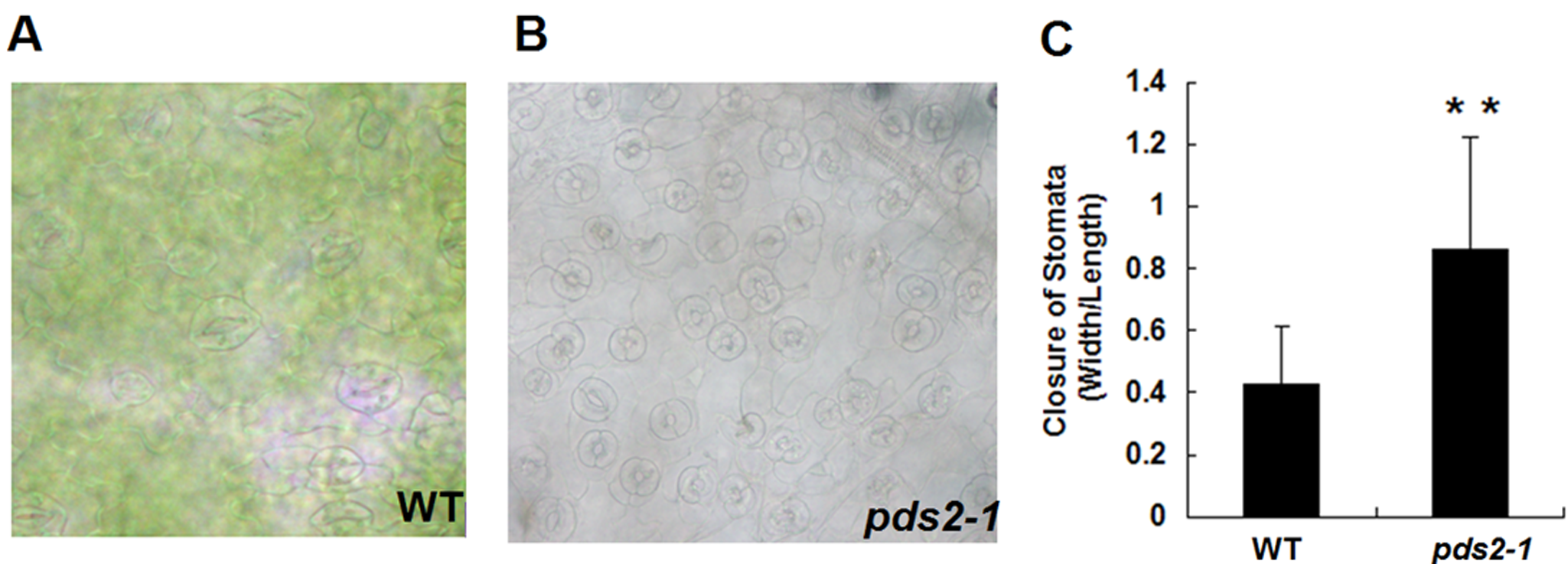

Supplement: Figure S4 — Microscopic and statistical analyses of stomata from the pds2-1 mutant and WT Arabidopsis. (TIF) [file pone.0094031.s004.tif]

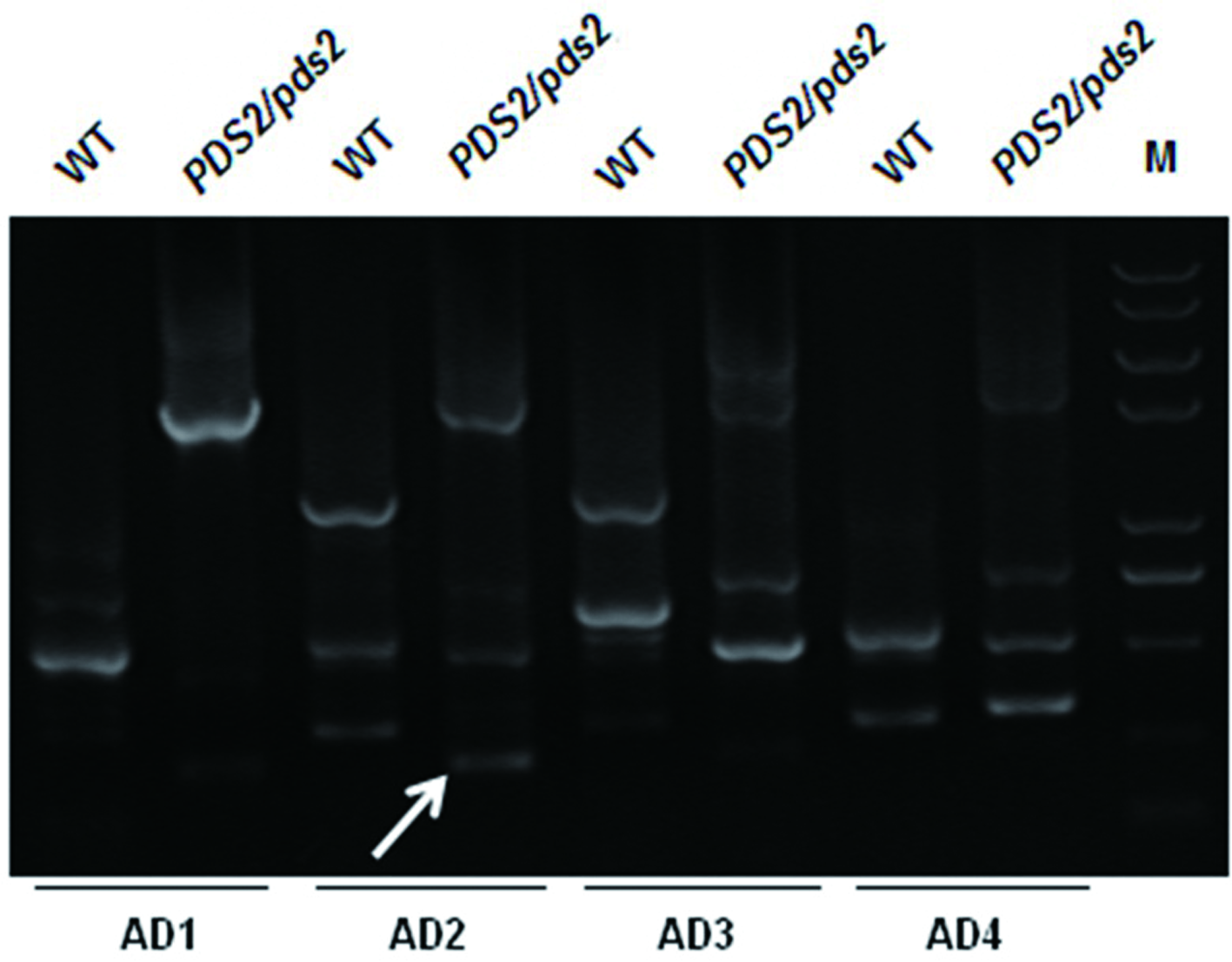

Supplement: Figure S5 — Representative TAIL-PCR analysis of heterozygous ( PDS2 / pds2 ) plants. (TIF) [file pone.0094031.s005.tif]

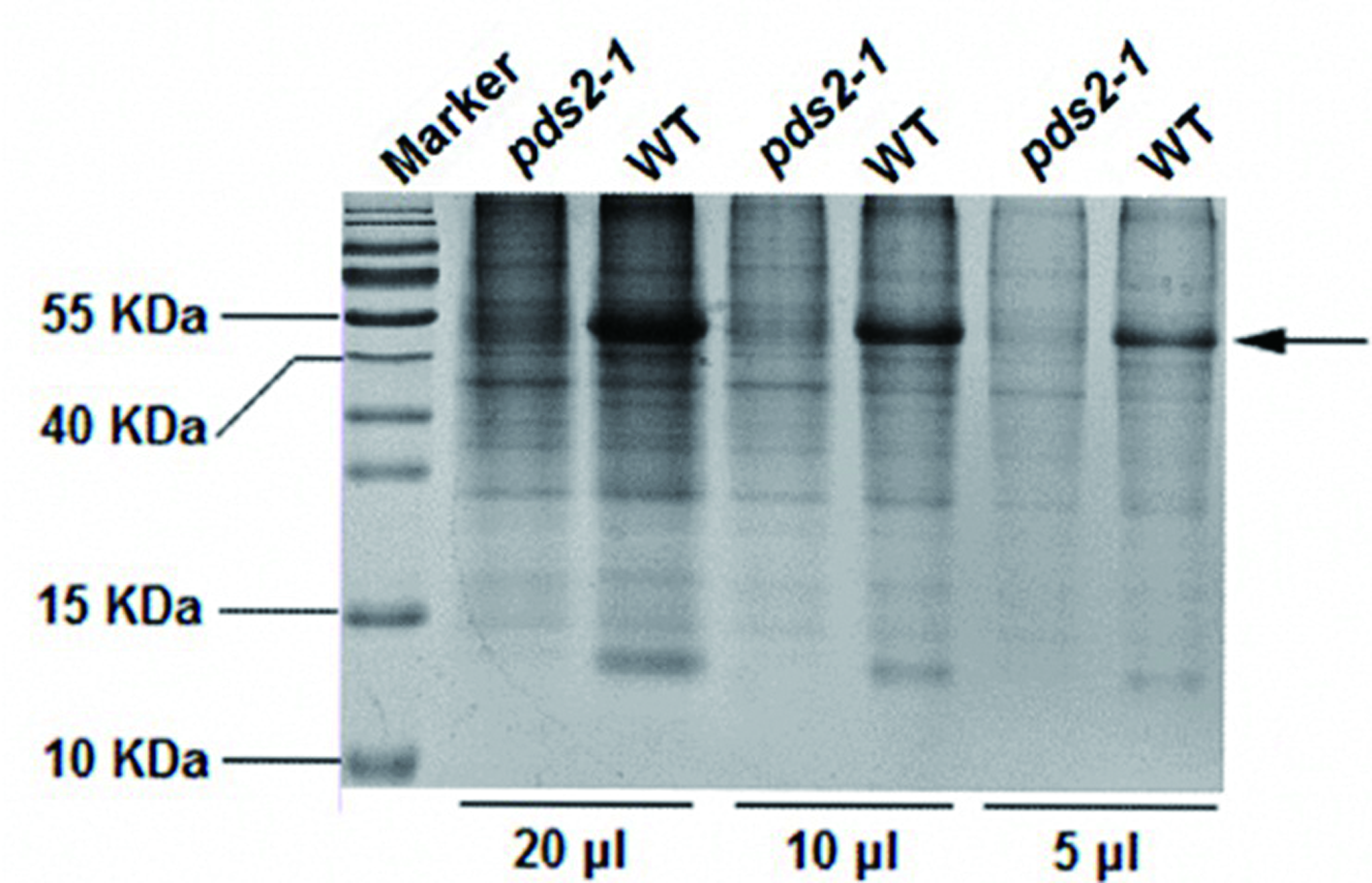

Supplement: Figure S6 — SDS-polyacrylamide gel electrophoresis showing loss of a major protein subunit (RuBisCo) in pds2-1 . (TIF) [file pone.0094031.s006.tif]

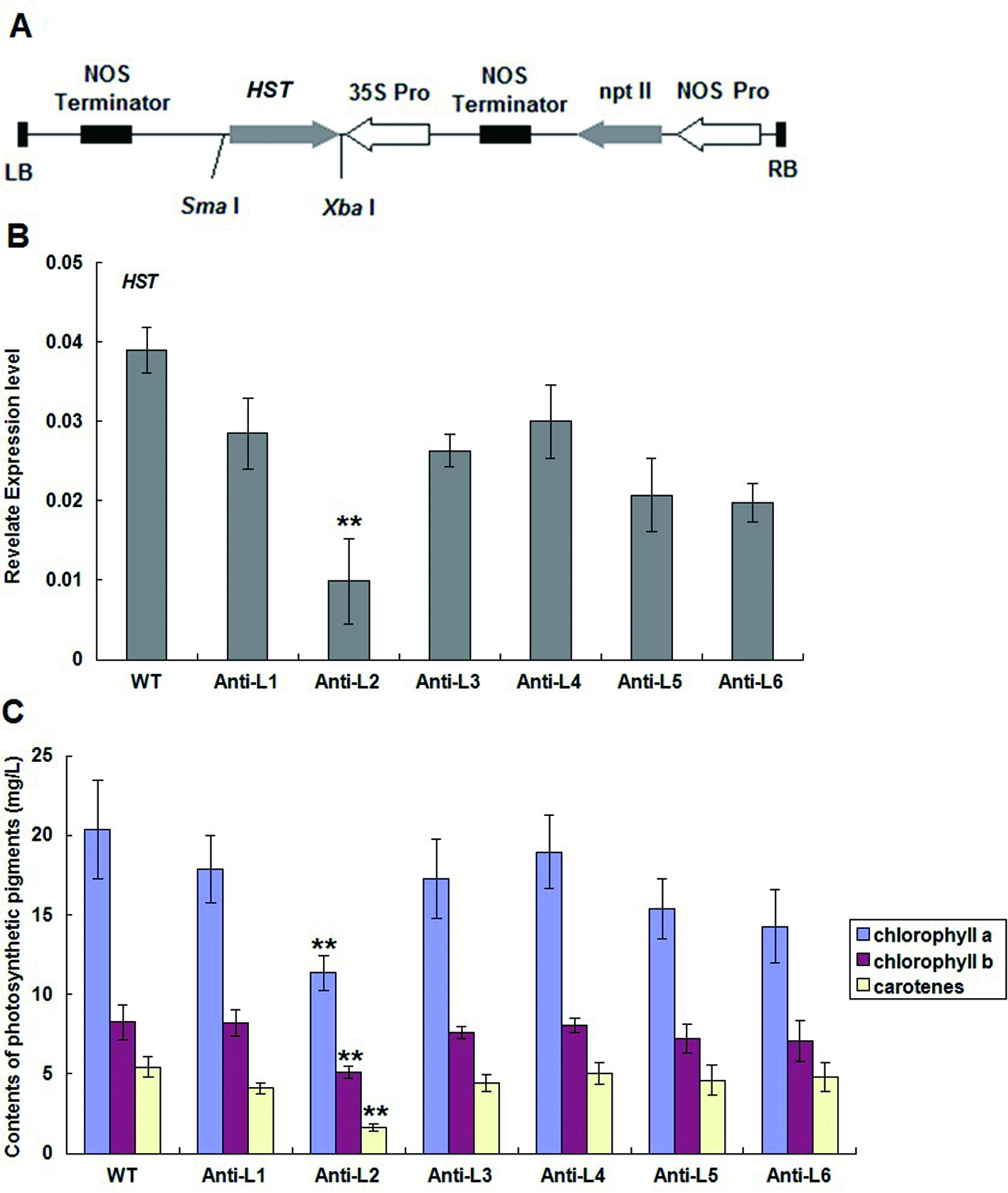

Supplement: Figure S7 — Gene expression and pigments in HST RNAi transgenic lines. (TIF) [file pone.0094031.s007.tif]
