# Supplementary material for: Disruption of the Homogentisate Solanesyltransferase Gene Results in Albino and Dwarf Phenotypes and Root, Trichome and Stomata Defects in Arabidopsis thaliana
Source: PLoS One. 2014 Apr 17;9(4):e94031. doi: 10.1371/journal.pone.0094031 (PMC3990575; doi:10.1371/journal.pone.0094031)
Supplement: Table S1 — Primers used in RT-qPCT analyses. (DOC) [file pone.0094031.s008.doc]

**Table S1** Primers used in RT-qPCT analyses.

| **Primer Name and type** | | **Sequence (5' to 3')** |
| --- | --- | --- |
| PDS1-1 | Forward | AAGTAGAAGACGCAGAGTCA |
| PDS1-2 | Reverse | ATCAGAGCCAGATGTTGTAG |
| ZDS-1 | Forward | AGATAGAGGTGGCAGAATCC |
| ZDS-2 | Reverse | GGTGTTAGAACGCACTGAAG |
| LYC-1 | Forward | TGGTCTGGTGCTGTTGTCTATG |
| LYC-2 | Reverse | GCTCGTCTTCCTCAATCCTCTT |
| PSY-1 | Forward | GAACCGAAGTAGAAGAATTG |
| PSY-2 | Reverse | GATCATCGAAGTTCTGGTAT |
| DXS-1 | Forward | GAGTATGCTCGAGGAATGATAA |
| DXS-2 | Reverse | AGACGAATAGATTGCACAGAAG |
| DXR-1 | Forward | ATGATGACATTAAACTCACTATCTCCAG |
| DXR-2 | Reverse | TCATGCATGAACTGGCCTAGCACCA |
| GA3-1 | Forward | ATGCGTTGACAGTCCTTACTTG |
| GA3-2 | Reverse | AACCATCTGCTTCAGTCGATCT |
| IM-1 | Forward | CCTTACAGACTCGGTTATTA |
| IM-2 | Reverse | TTACTGGTCTTCGAGTATTG |
| GL2-1 | Forward | CGGTGGTGTGACGATGATAC |
| GL2-2 | Reverse | GTGGAGATGAGCAGCGAGAA |
| WAVE1-1 | Forward | TATCTTGGACCGCTTCAACC |
| WAVE1-2 | Reverse | CGCACCATTGCTTCAGTTCT |
| GGPS-1 | Forward | ATCCACGAAGCGATGCGTTACT |
| GGPS-2 | Reverse | CACTTCCTCCACCAACAATAGC |
| GGRS-1 | Forward | CATCGAGCGTAAGATGGACAAT |
| GGRS-2 | Reverse | GCGACACATCATCTCCAACATA |
| ABA1-1 | Forward | GTCGGAAGCGAACCAGATCA |
| ABA1-2 | Reverse | ACGAGCATGCATCTTCGAAAC |
| GA1-1 | Forward | TTCATGCAGACCCGAGACAG |
| GA1-2 | Reverse | CCACGGGAAAGACATTGGGA |
| GA2-1 | Forward | ATCGGACCTCCACTTCCAGA |
| GA2-2 | Reverse | GAAACCGCATTCAGCTTCCC |
| WAVE4-1 | Forward | TGTTAGGCAACGACCATCAC |
| WAVE4-2 | Reverse | TTCAAGTCCAAGGCCAGTCT |
| FT-RT-f | Forward | GGAACAACCTTTGGCAATGAGAT |
| FT-RT-r | Reverse | CTGCCAAGCTGTCGAAACAA |
| GI-RT-f | Forward | CCCAAGTAGTGAGAATGACT |
| GI-RT-r | Reverse | CACCACTACACCATCGGAAA |
| CO-RT-f | Forward | AACTGCAGCGTACCACAGAC |
| CO-RT-r | Reverse | GGATGAAATGTATGCGTTATGG |
| SOC1-RT-f | Forward | GGATCGAGTCAGCACCAAACC |
| SOC1-RT-r | Reverse | CCCAATGAACAATTGCGTCTC |
| FLC-RT-f | Forward | ATCGATTCCGTTCTCGATGT |
| FLC-RT-r | Reverse | ATCCAGTTCCTCCTCCCAAC |
| TUB2-1 | Forward | GTTCTCGATGTTGTTCGTAAG |
| TUB2-2 | Reverse | TGTAAGGCTCAACCACAGTAT |
