# Supplementary material for: Disruption of the Homogentisate Solanesyltransferase Gene Results in Albino and Dwarf Phenotypes and Root, Trichome and Stomata Defects in Arabidopsis thaliana
Source: PLoS One. 2014 Apr 17;9(4):e94031. doi: 10.1371/journal.pone.0094031 (PMC3990575; doi:10.1371/journal.pone.0094031)
Supplement: Table S2 — Significant differential expression genes in WT and pds2-1 . (DOC) [file pone.0094031.s009.doc]

**Table S2** Significant differential expression genes in WT and *pds2-1*.

| **gene_id** | **gene** | **value_WT** | **value_pds2** | **log2(fold_change)** | **p_value** | **q_value** |
| --- | --- | --- | --- | --- | --- | --- |
| XLOC_010259 | LHB1B1 | 690.443 | 2.29618 | -8.23215 | 2.13E-11 | 1.47E-07 |
| XLOC_015264 | LHCB2.4 | 801.784 | 3.21811 | -7.96086 | 2.30E-11 | 1.47E-07 |
| XLOC_001607 | CAB1 | 15293.4 | 396.249 | -5.27036 | 1.34E-11 | 1.47E-07 |
| XLOC_003937 | AT1G75460 | 141.795 | 2.33388 | -5.92493 | 7.13E-11 | 3.41E-07 |
| XLOC_003800 | AT1G72910 | 102.834 | 0.613689 | -7.38859 | 2.24E-10 | 8.56E-07 |
| XLOC_020321 | AT3G63160 | 652.533 | 14.9384 | -5.44895 | 4.24E-10 | 1.35E-06 |
| XLOC_023744 | CYP706A6 | 74.8257 | 0.408739 | -7.51621 | 5.36E-10 | 1.46E-06 |
| XLOC_008018 | PETE1 | 277.748 | 7.50665 | -5.20946 | 6.62E-10 | 1.58E-06 |
| XLOC_005583 | AT1G25440 | 152.255 | 4.2749 | -5.15446 | 1.14E-09 | 2.43E-06 |
| XLOC_007851 | PS2 | 3.15927 | 122.344 | 5.27521 | 1.29E-09 | 2.47E-06 |
| XLOC_016935 | AT3G62550 | 161.008 | 2.10604 | -6.25645 | 1.73E-09 | 3.02E-06 |
| XLOC_021679 | NCED4 | 225.331 | 6.01658 | -5.22696 | 6.22E-09 | 9.32E-06 |
| XLOC_003687 | AT1G70820 | 113.448 | 4.21725 | -4.74958 | 6.33E-09 | 9.32E-06 |
| XLOC_030361 | VSP2 | 221.851 | 5.04776 | -5.4578 | 1.20E-08 | 1.64E-05 |
| XLOC_007625 | AT1G68520 | 79.5505 | 3.6989 | -4.4267 | 1.74E-08 | 2.22E-05 |
| XLOC_011832 | AT2G15020 | 32.9252 | 0.759842 | -5.43735 | 2.91E-08 | 3.48E-05 |
| XLOC_005380 | AT1G21500 | 449.29 | 21.4083 | -4.3914 | 3.71E-08 | 3.94E-05 |
| XLOC_025547 | ATIPS2 | 23.4088 | 460.15 | 4.29699 | 3.60E-08 | 3.94E-05 |
| XLOC_021378 | ACD6 | 109.869 | 4.29591 | -4.67668 | 5.63E-08 | 5.67E-05 |
| XLOC_013434 | COR15B | 87.0479 | 1.43487 | -5.92282 | 6.32E-08 | 5.75E-05 |
| XLOC_004343 | PSAD-2 | 984.828 | 17.2276 | -5.83708 | 6.26E-08 | 5.75E-05 |
| XLOC_025260 | NDH-M | 113.126 | 5.02901 | -4.49151 | 8.44E-08 | 7.34E-05 |
| XLOC_019916 | WRKY70 | 161.528 | 10.723 | -3.913 | 2.01E-07 | 0.000166696 |
| XLOC_015160 | AOC2 | 225.157 | 14.7749 | -3.92971 | 2.30E-07 | 0.000183276 |
| XLOC_007884 | NUDT21 | 107.957 | 6.82177 | -3.98417 | 2.75E-07 | 0.00020191 |
| XLOC_001016 | LHCA6 | 185.363 | 12.8615 | -3.84922 | 2.72E-07 | 0.00020191 |
| XLOC_001287 | AT1G24147 | 98.6278 | 1.37016 | -6.16958 | 3.06E-07 | 0.000209153 |
| XLOC_012021 | AT2G18193 | 3.90114 | 58.3254 | 3.90216 | 3.04E-07 | 0.000209153 |
| XLOC_031123 | AT5G38420 | 2845.75 | 3.63957 | -9.61082 | 3.67E-07 | 0.000236591 |
| XLOC_004576 | AT1G07135 | 105.732 | 6.81754 | -3.95502 | 3.84E-07 | 0.000236591 |
| XLOC_021991 | DIC2 | 85.5132 | 6.07027 | -3.81631 | 3.79E-07 | 0.000236591 |
| XLOC_016927 | CP12 | 764.087 | 29.2305 | -4.70819 | 4.86E-07 | 0.000290262 |
| XLOC_019184 | AT3G44260 | 131.195 | 10.0374 | -3.70826 | 5.14E-07 | 0.000297969 |
| XLOC_032056 | ANK | 43.0207 | 1.65692 | -4.69845 | 5.36E-07 | 0.000301506 |
| XLOC_003802 | TIR | 292.083 | 15.9518 | -4.19459 | 6.17E-07 | 0.000336998 |
| XLOC_005773 | COR414-TM1 | 53.8154 | 1.06505 | -5.65903 | 6.96E-07 | 0.000369578 |
| XLOC_022098 | AT4G26530 | 670.479 | 1.87028 | -8.4858 | 7.38E-07 | 0.000381395 |
| XLOC_009520 | AT2G21640 | 5.33115 | 110.975 | 4.37964 | 8.12E-07 | 0.000408444 |
| XLOC_009615 | SCPL13 | 44.4018 | 1.37971 | -5.00818 | 8.93E-07 | 0.000437834 |
| XLOC_021325 | AT4G13495 | 146.419 | 2.41766 | -5.92035 | 1.02E-06 | 0.000473776 |
| XLOC_008069 | AT1G76960 | 221.076 | 10.6637 | -4.37376 | 1.01E-06 | 0.000473776 |
| XLOC_018210 | PCC1 | 101.875 | 1.06279 | -6.58279 | 1.08E-06 | 0.000492826 |
| XLOC_007199 | AT1G61300 | 23.9657 | 1.50065 | -3.99732 | 1.22E-06 | 0.000529575 |
| XLOC_003402 | ARK2 | 45.9542 | 3.9321 | -3.54682 | 1.22E-06 | 0.000529575 |
| XLOC_032984 | PSBA | 67.8393 | 3.07681 | -4.46261 | 1.25E-06 | 0.000530008 |
| XLOC_010267 | AT2G34620 | 43.2943 | 1.12466 | -5.26661 | 1.41E-06 | 0.000585198 |
| XLOC_011215 | AT2G04050 | 6.7312 | 78.6176 | 3.54592 | 1.76E-06 | 0.000714151 |
| XLOC_013435 | COR15 | 109.566 | 7.02384 | -3.9634 | 1.85E-06 | 0.000738157 |
| XLOC_013530 | AT2G44290 | 70.3843 | 4.91668 | -3.8395 | 1.89E-06 | 0.000739466 |
| XLOC_000725 | PQL1 | 137.711 | 7.28368 | -4.24083 | 2.02E-06 | 0.000770656 |
| XLOC_023718 | AT4G11900 | 17.443 | 0.475522 | -5.19699 | 2.32E-06 | 0.000820993 |
| XLOC_015303 | AT3G28220 | 34.0191 | 1.29727 | -4.7128 | 2.22E-06 | 0.000820993 |
| XLOC_005185 | AT1G17960 | 1.17571 | 26.2153 | 4.4788 | 2.29E-06 | 0.000820993 |
| XLOC_028180 | AT5G51440 | 1.41219 | 38.5177 | 4.76951 | 2.26E-06 | 0.000820993 |
| XLOC_025458 | AT5G02160 | 668.924 | 39.1525 | -4.09467 | 2.50E-06 | 0.000868347 |
| XLOC_018211 | AT3G22235 | 562.217 | 31.611 | -4.15263 | 2.84E-06 | 0.000970319 |
| XLOC_003801 | AT1G72920 | 35.23 | 1.28123 | -4.78121 | 2.91E-06 | 0.000975128 |
| XLOC_011192 | AT2G03750 | 41.2503 | 2.48086 | -4.05549 | 3.19E-06 | 0.0010343 |
| XLOC_010115 | AT2G32160 | 62.903 | 5.80613 | -3.43748 | 3.19E-06 | 0.0010343 |
| XLOC_023835 | AT4G13500 | 171.812 | 15.4406 | -3.47602 | 3.33E-06 | 0.00106189 |
| XLOC_018181 | ICL | 2.13333 | 25.0027 | 3.5509 | 3.39E-06 | 0.00106359 |
| XLOC_013494 | AT2G43620 | 10.0669 | 104.057 | 3.36968 | 3.55E-06 | 0.00109437 |
| XLOC_031800 | ATFRO6 | 388.71 | 13.8392 | -4.81187 | 3.77E-06 | 0.00112569 |
| XLOC_025360 | FKBP16-2 | 141.683 | 12.9198 | -3.45501 | 3.75E-06 | 0.00112569 |
| XLOC_009961 | AT2G29290 | 123.085 | 5.44334 | -4.49902 | 3.89E-06 | 0.00114334 |
| XLOC_031801 | ATFRO7 | 161.056 | 9.18344 | -4.13239 | 4.14E-06 | 0.0011994 |
| XLOC_019859 | AT3G55240 | 58.7371 | 0.598579 | -6.61659 | 4.62E-06 | 0.00131784 |
| XLOC_027763 | GLK2 | 128.605 | 12.4273 | -3.37137 | 5.30E-06 | 0.00148891 |
| XLOC_002689 | AT1G52870 | 174.031 | 16.813 | -3.3717 | 5.65E-06 | 0.00156692 |
| XLOC_005686 | STZ | 146.911 | 15.5581 | -3.2392 | 6.20E-06 | 0.00169247 |
| XLOC_031726 | AT5G48490 | 92.3721 | 1.59919 | -5.85205 | 6.49E-06 | 0.00174732 |
| XLOC_017613 | ATHST | 33.8317 | 1.00293 | -5.07608 | 6.76E-06 | 0.00179641 |
| XLOC_015965 | FAMT | 35.265 | 0.150341 | -7.87386 | 7.28E-06 | 0.0019063 |
| XLOC_026466 | AT5G21430 | 93.8726 | 2.76317 | -5.08631 | 9.57E-06 | 0.0024306 |
| XLOC_031468 | AT5G44260 | 37.5568 | 3.25448 | -3.52858 | 9.63E-06 | 0.0024306 |
| XLOC_028197 | AT5G51720 | 365.851 | 39.3029 | -3.21855 | 9.66E-06 | 0.0024306 |
| XLOC_003964 | ATA27 | 0.493324 | 14.7049 | 4.89762 | 1.00E-05 | 0.0024835 |
| XLOC_013306 | PYL6 | 9.93089 | 89.2592 | 3.16801 | 1.02E-05 | 0.00249889 |
| XLOC_014193 | IPS1 | 0.7207 | 92.5641 | 7.00491 | 1.05E-05 | 0.00254005 |
| XLOC_027794 | AT5G44580 | 367.043 | 42.9349 | -3.09573 | 1.12E-05 | 0.00267037 |
| XLOC_020120 | AT3G59930 | 1.87177 | 50.981 | 4.76748 | 1.39E-05 | 0.00327173 |
| XLOC_009189 | AT2G15960 | 235.564 | 24.8713 | -3.24357 | 1.50E-05 | 0.00346691 |
| XLOC_030327 | GMI1 | 1.17716 | 11.5271 | 3.29166 | 1.50E-05 | 0.00346691 |
| XLOC_017656 | HSP70 | 7.67076 | 67.3499 | 3.13424 | 1.52E-05 | 0.00346878 |
| XLOC_004352 | AT1G03400 | 43.0896 | 4.43159 | -3.28144 | 1.63E-05 | 0.00366496 |
| XLOC_019661 | SULTR3 | 46.3066 | 5.63497 | -3.03874 | 1.69E-05 | 0.00375877 |
| XLOC_029228 | AT5G03350 | 73.6592 | 7.88612 | -3.22348 | 1.74E-05 | 0.00382372 |
| XLOC_030149 | GLP3A | 374.448 | 40.1543 | -3.22114 | 1.86E-05 | 0.00404144 |
| XLOC_027948 | atnudt8 | 27.1324 | 1.78132 | -3.929 | 1.98E-05 | 0.00426021 |
| XLOC_028703 | AT5G60250 | 0.997639 | 14.3825 | 3.84966 | 2.06E-05 | 0.00436896 |
| XLOC_000989 | NDF6 | 206.294 | 13.6674 | -3.91589 | 2.13E-05 | 0.00447637 |
| XLOC_010140 | ATCSLB4 | 11.3179 | 0.58018 | -4.28596 | 2.95E-05 | 0.00613186 |
| XLOC_012652 | CYP707A2 | 19.1222 | 0.365311 | -5.70998 | 3.21E-05 | 0.00643037 |
| XLOC_026486 | NIT4 | 54.5129 | 6.97602 | -2.96612 | 3.23E-05 | 0.00643037 |
| XLOC_018179 | AT3G21670 | 50.7682 | 6.78124 | -2.9043 | 3.21E-05 | 0.00643037 |
| XLOC_031370 | AOS | 66.2384 | 8.89379 | -2.8968 | 3.20E-05 | 0.00643037 |
| XLOC_029916 | TRXF2 | 209.817 | 27.9653 | -2.90742 | 3.96E-05 | 0.00780164 |
| XLOC_010304 | AT2G35260 | 198.961 | 21.9422 | -3.18071 | 4.14E-05 | 0.00808408 |
| XLOC_016184 | AT3G48650 | 25.9865 | 1.60817 | -4.01427 | 4.35E-05 | 0.00837793 |
| XLOC_001422 | AT1G27020 | 18.8787 | 141.211 | 2.90303 | 4.38E-05 | 0.00837793 |
| XLOC_026412 | ATSPX1 | 22.2151 | 162.775 | 2.87327 | 4.46E-05 | 0.00843431 |
| XLOC_031018 | AT5G36790 | 145.038 | 11.7008 | -3.63175 | 4.50E-05 | 0.00843818 |
| XLOC_004942 | AT1G13470 | 29.6724 | 3.0361 | -3.28883 | 4.59E-05 | 0.00844248 |
| XLOC_032201 | AT5G57345 | 354.723 | 44.2596 | -3.00263 | 4.59E-05 | 0.00844248 |
| XLOC_004042 | TAPX | 74.3075 | 10.5882 | -2.81105 | 5.03E-05 | 0.00916273 |
| XLOC_020425 | AT4G01460 | 17.0721 | 0.953073 | -4.16291 | 5.09E-05 | 0.00917719 |
| XLOC_009458 | GPRI1 | 52.5799 | 4.7305 | -3.47444 | 5.36E-05 | 0.00958654 |
| XLOC_019297 | CRK4 | 18.4893 | 0.274723 | -6.07257 | 5.51E-05 | 0.00965892 |
| XLOC_023826 | DVL10 | 67.0728 | 2.2484 | -4.89875 | 5.49E-05 | 0.00965892 |
| XLOC_024955 | AT4G32340 | 79.2013 | 11.2751 | -2.81239 | 6.19E-05 | 0.0106618 |
| XLOC_008930 | MGDC | 0.1472 | 21.3919 | 7.18315 | 6.19E-05 | 0.0106618 |
| XLOC_011026 | AT2G47880 | 36.7828 | 0.664997 | -5.78954 | 6.75E-05 | 0.0113219 |
| XLOC_031665 | ATTIP2 | 19.1785 | 0.51835 | -5.20942 | 6.71E-05 | 0.0113219 |
| XLOC_022496 | AT4G33550 | 90.3323 | 7.02141 | -3.68541 | 6.74E-05 | 0.0113219 |
| XLOC_021895 | AT4G22960 | 0.142729 | 11.6357 | 6.34913 | 6.95E-05 | 0.0115501 |
| XLOC_033054 | RRN16S.2 | 129.498 | 15.4715 | -3.06524 | 7.18E-05 | 0.0117076 |
| XLOC_031572 | CIPK20 | 34.1191 | 4.40267 | -2.95413 | 7.29E-05 | 0.0117076 |
| XLOC_021288 | AT4G12870 | 13.346 | 98.6113 | 2.88534 | 7.14E-05 | 0.0117076 |
| XLOC_005540 | SEP3 | 1.41157 | 24.9518 | 4.14378 | 7.23E-05 | 0.0117076 |
| XLOC_008323 | MLP328 | 87.4725 | 12.3436 | -2.82506 | 7.39E-05 | 0.0117253 |
| XLOC_032440 | ERF104 | 77.1859 | 11.5472 | -2.7408 | 7.42E-05 | 0.0117253 |
| XLOC_004768 | ATGSTU18 | 65.8753 | 8.80551 | -2.90326 | 7.53E-05 | 0.0117399 |
| XLOC_000600 | AT1G12010 | 3.24326 | 28.8241 | 3.15176 | 7.55E-05 | 0.0117399 |
| XLOC_005383 | AT1G21525 | 2.31879 | 49.2248 | 4.40794 | 7.78E-05 | 0.0119983 |
| XLOC_028812 | AT5G62280 | 33.8445 | 3.66201 | -3.20822 | 7.99E-05 | 0.0122161 |
| XLOC_032947 | RBCL | 20.5372 | 1.7385 | -3.56232 | 8.87E-05 | 0.013432 |
| XLOC_021215 | CRK30 | 0.696248 | 12.3308 | 4.14653 | 8.92E-05 | 0.013432 |
| XLOC_022146 | PORB | 644.391 | 23.545 | -4.77444 | 0.000101662 | 0.0150688 |
| XLOC_032964 | RRN16S.1 | 129.498 | 16.185 | -3.0002 | 0.000101545 | 0.0150688 |
| XLOC_007724 | AT1G70260 | 22.6085 | 2.10082 | -3.42784 | 0.000104159 | 0.0151124 |
| XLOC_000961 | NUDT4 | 43.1196 | 5.50847 | -2.96862 | 0.000104327 | 0.0151124 |
| XLOC_030319 | SQP1 | 33.0077 | 4.71794 | -2.80657 | 0.000103285 | 0.0151124 |
| XLOC_016953 | AT3G62950 | 30.7549 | 0.910022 | -5.07877 | 0.000106062 | 0.0151344 |
| XLOC_024666 | AT4G27280 | 133.865 | 20.8667 | -2.68151 | 0.000105439 | 0.0151344 |
| XLOC_014264 | LBD21 | 70.2426 | 7.83129 | -3.16503 | 0.000113275 | 0.0159259 |
| XLOC_029503 | FTRA2 | 180.728 | 28.6559 | -2.65692 | 0.000112824 | 0.0159259 |
| XLOC_008326 | CRR3 | 70.3037 | 10.0533 | -2.80594 | 0.000116795 | 0.016301 |
| XLOC_010866 | AT2G45135 | 1.32641 | 47.5432 | 5.16364 | 0.000118427 | 0.016409 |
| XLOC_031190 | AT5G39530 | 79.0025 | 12.7926 | -2.62659 | 0.000125031 | 0.0171994 |
| XLOC_019932 | SIB1 | 101.612 | 15.1317 | -2.74743 | 0.000127793 | 0.0174538 |
| XLOC_028250 | AtHsp90-1 | 4.57565 | 28.4629 | 2.63703 | 0.000128929 | 0.0174841 |
| XLOC_000822 | NDF1 | 205.166 | 23.991 | -3.09623 | 0.000130798 | 0.0176126 |
| XLOC_031054 | AT5G37360 | 141.505 | 22.192 | -2.67275 | 0.000132942 | 0.0177761 |
| XLOC_029620 | AT5G10760 | 29.567 | 4.16539 | -2.82746 | 0.000136346 | 0.0181047 |
| XLOC_032568 | anac103 | 0.333707 | 11.2287 | 5.07247 | 0.000138067 | 0.0182067 |
| XLOC_019643 | LTP12 | 3.55263 | 47.64 | 3.74521 | 0.0001396 | 0.0182828 |
| XLOC_031009 | ATPGLP1 | 274.483 | 19.3265 | -3.82807 | 0.000148142 | 0.0192695 |
| XLOC_007912 | AT1G74070 | 38.2053 | 5.89259 | -2.6968 | 0.000149558 | 0.0193222 |
| XLOC_005029 | PCR1 | 24.1701 | 0.523209 | -5.52969 | 0.000150899 | 0.0193647 |
| XLOC_010575 | LHCB4.3 | 91.2634 | 13.8916 | -2.71583 | 0.000155209 | 0.019785 |
| XLOC_026632 | CYP71B12 | 12.9284 | 0.864534 | -3.90247 | 0.000158482 | 0.0200684 |
| XLOC_000456 | AT1G09500 | 29.4717 | 2.2035 | -3.74147 | 0.000172738 | 0.0217298 |
| XLOC_018195 | CYCP2 | 26.2781 | 0.265741 | -6.6277 | 0.000174421 | 0.0217981 |
| XLOC_025582 | PKS4 | 14.5043 | 1.31792 | -3.46014 | 0.00018566 | 0.023052 |
| XLOC_012870 | AT2G32870 | 19.2331 | 2.06335 | -3.22053 | 0.000190322 | 0.0234783 |
| XLOC_007767 | ATMYBL2 | 191.261 | 32.1513 | -2.57259 | 0.000197296 | 0.0241827 |
| XLOC_013465 | AT2G43140 | 3.92862 | 48.8864 | 3.63734 | 0.000199567 | 0.0243053 |
| XLOC_023572 | AT4G09350 | 23.4301 | 1.75782 | -3.73651 | 0.000206148 | 0.0247909 |
| XLOC_008019 | AT1G76110 | 28.2482 | 3.82967 | -2.88287 | 0.000205922 | 0.0247909 |
| XLOC_026120 | AT5G14970 | 120.415 | 20.5173 | -2.5531 | 0.000209973 | 0.0249372 |
| XLOC_005617 | AT1G26390 | 1.61038 | 14.64 | 3.18444 | 0.000209039 | 0.0249372 |
| XLOC_031100 | AT5G37990 | 25.2298 | 2.94946 | -3.09661 | 0.000212356 | 0.0250645 |
| XLOC_002652 | PSAH2 | 1706.58 | 99.4157 | -4.10149 | 0.000235681 | 0.027647 |
| XLOC_003401 | ARK1 | 6.01898 | 0.366298 | -4.03843 | 0.000239443 | 0.0279171 |
| XLOC_004486 | chr31 | 0.0818281 | 2.44193 | 4.89928 | 0.000243498 | 0.0282177 |
| XLOC_007424 | AT1G65190 | 29.9345 | 4.5046 | -2.73234 | 0.000247119 | 0.0284648 |
| XLOC_009096 | LURP1 | 283.226 | 25.7088 | -3.46162 | 0.000255128 | 0.0289411 |
| XLOC_032302 | AT5G59080 | 88.5585 | 14.5773 | -2.60291 | 0.000257308 | 0.0289411 |
| XLOC_027435 | AT5G38940 | 9.57369 | 61.777 | 2.68992 | 0.000256113 | 0.0289411 |
| XLOC_005170 | AT1G17710 | 0.253672 | 60.3973 | 7.89538 | 0.000255395 | 0.0289411 |
| XLOC_021899 | IQD22 | 22.0152 | 3.30408 | -2.73618 | 0.000270659 | 0.0302647 |
| XLOC_020090 | GUN4 | 129.063 | 22.859 | -2.49724 | 0.000281728 | 0.0313193 |
| XLOC_031565 | AT5G45650 | 51.4583 | 9.13796 | -2.49346 | 0.000287828 | 0.0318125 |
| XLOC_032254 | AT5G58260 | 119.153 | 16.8507 | -2.82193 | 0.000293674 | 0.0320877 |
| XLOC_014724 | AT3G18890 | 105.631 | 15.9574 | -2.72673 | 0.000292165 | 0.0320877 |
| XLOC_002249 | AT1G43670 | 395.034 | 40.9848 | -3.26882 | 0.000305989 | 0.0332433 |
| XLOC_016467 | Fes1B | 55.4664 | 9.96838 | -2.47618 | 0.000311475 | 0.0336481 |
| XLOC_029736 | SAG29 | 18.3115 | 1.47036 | -3.63851 | 0.00031505 | 0.0338431 |
| XLOC_021141 | AT4G10300 | 184.462 | 33.7713 | -2.44945 | 0.000320209 | 0.0342051 |
| XLOC_016069 | PTAC16 | 465.926 | 22.2496 | -4.38825 | 0.000327796 | 0.0348211 |
| XLOC_002568 | AT1G50732 | 54.3154 | 4.44631 | -3.61068 | 0.000350169 | 0.0369922 |
| XLOC_012343 | AT2G23840 | 52.8189 | 9.0768 | -2.5408 | 0.000354279 | 0.0372207 |
| XLOC_015126 | AT3G25240 | 0.267424 | 14.2707 | 5.73778 | 0.000363019 | 0.0379306 |
| XLOC_002805 | AT1G54820 | 27.538 | 4.16552 | -2.72486 | 0.000366612 | 0.0380977 |
| XLOC_024097 | ATERF6 | 26.0608 | 3.8368 | -2.76391 | 0.000375756 | 0.0388369 |
| XLOC_015024 | AT3G23480 | 18.0499 | 1.80401 | -3.32271 | 0.000378785 | 0.0389395 |
| XLOC_032160 | ATOXS3 | 36.6886 | 3.81076 | -3.26718 | 0.000384814 | 0.0393142 |
| XLOC_017049 | SRG3 | 9.08079 | 58.0776 | 2.67709 | 0.000386542 | 0.0393142 |
| XLOC_017882 | PSAH-1 | 1729.64 | 98.715 | -4.13106 | 0.00039047 | 0.0395036 |
| XLOC_011301 | AT2G05310 | 341.468 | 62.7366 | -2.44437 | 0.000418232 | 0.0420895 |
| XLOC_026181 | KIN1 | 67.3539 | 10.4314 | -2.69083 | 0.000434818 | 0.043251 |
| XLOC_007888 | AT1G73655 | 193.339 | 34.7235 | -2.47715 | 0.000436559 | 0.043251 |
| XLOC_010645 | M17 | 0.302496 | 11.1698 | 5.20655 | 0.000434074 | 0.043251 |
| XLOC_011280 | LHCB2 | 2396.64 | 20.3789 | -6.8778 | 0.000442562 | 0.0436197 |
| XLOC_010451 | AT2G37870 | 35.1376 | 2.58082 | -3.76711 | 0.000447149 | 0.0436221 |
| XLOC_021571 | TIP2 | 218.155 | 38.499 | -2.50246 | 0.000446202 | 0.0436221 |
| XLOC_030261 | CaS | 394.599 | 43.0824 | -3.19522 | 0.00044983 | 0.0436609 |
| XLOC_016589 | ATSZF1 | 71.1686 | 12.9249 | -2.46109 | 0.000455643 | 0.0440018 |
| XLOC_012904 | CLO-3 | 67.8071 | 11.7916 | -2.52368 | 0.000461928 | 0.0442328 |
| XLOC_015297 | AT3G28160 | 4.54735 | 25.3385 | 2.47823 | 0.000462662 | 0.0442328 |
| XLOC_017080 | TRXF1 | 558.484 | 75.5763 | -2.88551 | 0.000469449 | 0.0445011 |
| XLOC_003803 | AT1G72940 | 28.4097 | 4.635 | -2.61574 | 0.000471433 | 0.0445011 |
| XLOC_013597 | AGL20 | 45.1821 | 8.06837 | -2.4854 | 0.00047245 | 0.0445011 |
| XLOC_001207 | AGL87 | 25.5439 | 1.23593 | -4.36931 | 0.000486877 | 0.0454639 |
| XLOC_022293 | AT4G29780 | 20.5521 | 3.65935 | -2.48963 | 0.000487427 | 0.0454639 |
| XLOC_032308 | AT5G59130 | 17.7572 | 1.31411 | -3.75625 | 0.000505527 | 0.0466966 |
| XLOC_000738 | AT1G14345 | 102.763 | 19.3784 | -2.4068 | 0.000503661 | 0.0466966 |
| XLOC_006745 | AT1G52220 | 618.499 | 75.9158 | -3.0263 | 0.000511372 | 0.0469572 |
| XLOC_026077 | CARAB-AK-LYS | 75.8446 | 11.0749 | -2.77575 | 0.000513261 | 0.0469572 |
| XLOC_021286 | AT4G12830 | 30.7414 | 1.36757 | -4.49049 | 0.000519879 | 0.0473362 |
| XLOC_017169 | AT3G04210 | 41.8565 | 8.19751 | -2.35219 | 0.000523652 | 0.0474537 |
| XLOC_019963 | BGL2 | 25.8934 | 4.32508 | -2.58179 | 0.000531124 | 0.0479039 |
| XLOC_031362 | AT5G42530 | 3573.74 | 191.774 | -4.21995 | 0.000542275 | 0.0484326 |
| XLOC_024057 | AT4G16880 | 59.5937 | 10.9821 | -2.44 | 0.000544585 | 0.0484326 |
| XLOC_020021 | AT3G58270 | 2.19791 | 19.6641 | 3.16136 | 0.000540153 | 0.0484326 |
| XLOC_003368 | AT1G65230 | 106.997 | 16.1549 | -2.72753 | 0.000553552 | 0.0485526 |
| XLOC_023787 | AT4G12980 | 49.3365 | 9.63695 | -2.35601 | 0.000552648 | 0.0485526 |
| XLOC_032765 | AT5G67370 | 67.4428 | 13.41 | -2.33035 | 0.00055085 | 0.0485526 |
| XLOC_009440 | PSAE-2 | 1120.81 | 77.3941 | -3.85618 | 0.000557058 | 0.048637 |
| XLOC_029960 | PSY | 149.879 | 22.8618 | -2.71279 | 0.000568601 | 0.0491955 |
| XLOC_024322 | ARK3 | 14.7537 | 2.63354 | -2.48601 | 0.000566609 | 0.0491955 |
| XLOC_012753 | CYP71A12 | 11.2534 | 56.2695 | 2.32199 | 0.000578139 | 0.0497955 |
