# Supplementary material for: Disruption of the Homogentisate Solanesyltransferase Gene Results in Albino and Dwarf Phenotypes and Root, Trichome and Stomata Defects in Arabidopsis thaliana
Source: PLoS One. 2014 Apr 17;9(4):e94031. doi: 10.1371/journal.pone.0094031 (PMC3990575; doi:10.1371/journal.pone.0094031)
Supplement: Table S3 — GO terms in all three categories (Biological Process, Cellular Component and Mollecular Function) generated by SEA. (DOC) [file pone.0094031.s010.doc]

**Table S3** GO terms in all three categories (Biological Process, Cellular Component and Mollecular Function) generated by SEA.

| **GO term** | **Ontology** | **Description** | **Number in input list** | **Number in BG/Ref** | **p-value** | **FDR** |
| --- | --- | --- | --- | --- | --- | --- |
| GO:0050896 | P | response to stimulus | [55](http://bioinfo.cau.edu.cn/agriGO/termDetail.php?session=841659544&GO=GO:0050896) | 985 | 5.80E-30 | 3.50E-27 |
| GO:0006950 | P | response to stress | [38](http://bioinfo.cau.edu.cn/agriGO/termDetail.php?session=841659544&GO=GO:0006950) | 456 | 9.30E-28 | 2.80E-25 |
| GO:0015979 | P | photosynthesis | [16](http://bioinfo.cau.edu.cn/agriGO/termDetail.php?session=841659544&GO=GO:0015979) | 39 | 9.10E-25 | 1.80E-22 |
| GO:0006952 | P | defense response | [15](http://bioinfo.cau.edu.cn/agriGO/termDetail.php?session=841659544&GO=GO:0006952) | 82 | 2.50E-17 | 2.90E-15 |
| GO:0009266 | P | response to temperature stimulus | [13](http://bioinfo.cau.edu.cn/agriGO/termDetail.php?session=841659544&GO=GO:0009266) | 51 | 2.90E-17 | 2.90E-15 |
| GO:0009628 | P | response to abiotic stimulus | [24](http://bioinfo.cau.edu.cn/agriGO/termDetail.php?session=841659544&GO=GO:0009628) | 318 | 2.10E-17 | 2.90E-15 |
| GO:0009409 | P | response to cold | [10](http://bioinfo.cau.edu.cn/agriGO/termDetail.php?session=841659544&GO=GO:0009409) | 28 | 2.60E-15 | 2.30E-13 |
| GO:0042221 | P | response to chemical stimulus | [24](http://bioinfo.cau.edu.cn/agriGO/termDetail.php?session=841659544&GO=GO:0042221) | 439 | 2.60E-14 | 2.00E-12 |
| GO:0065007 | P | biological regulation | [32](http://bioinfo.cau.edu.cn/agriGO/termDetail.php?session=841659544&GO=GO:0065007) | 882 | 1.80E-13 | 1.20E-11 |
| GO:0009751 | P | response to salicylic acid stimulus | [6](http://bioinfo.cau.edu.cn/agriGO/termDetail.php?session=841659544&GO=GO:0009751) | 7 | 6.70E-13 | 4.10E-11 |
| GO:0009605 | P | response to external stimulus | [11](http://bioinfo.cau.edu.cn/agriGO/termDetail.php?session=841659544&GO=GO:0009605) | 65 | 9.80E-13 | 5.40E-11 |
| GO:0010033 | P | response to organic substance | [16](http://bioinfo.cau.edu.cn/agriGO/termDetail.php?session=841659544&GO=GO:0010033) | 210 | 2.90E-12 | 1.40E-10 |
| GO:0043170 | P | macromolecule metabolic process | [32](http://bioinfo.cau.edu.cn/agriGO/termDetail.php?session=841659544&GO=GO:0043170) | 983 | 2.90E-12 | 1.40E-10 |
| GO:0051707 | P | response to other organism | [12](http://bioinfo.cau.edu.cn/agriGO/termDetail.php?session=841659544&GO=GO:0051707) | 96 | 4.00E-12 | 1.60E-10 |
| GO:0050794 | P | regulation of cellular process | [26](http://bioinfo.cau.edu.cn/agriGO/termDetail.php?session=841659544&GO=GO:0050794) | 658 | 3.90E-12 | 1.60E-10 |
| GO:0050789 | P | regulation of biological process | [27](http://bioinfo.cau.edu.cn/agriGO/termDetail.php?session=841659544&GO=GO:0050789) | 767 | 2.10E-11 | 7.90E-10 |
| GO:0009058 | P | biosynthetic process | [28](http://bioinfo.cau.edu.cn/agriGO/termDetail.php?session=841659544&GO=GO:0009058) | 834 | 2.70E-11 | 9.70E-10 |
| GO:0044260 | P | cellular macromolecule metabolic process | [27](http://bioinfo.cau.edu.cn/agriGO/termDetail.php?session=841659544&GO=GO:0044260) | 888 | 4.90E-10 | 1.70E-08 |
| GO:0045449 | P | regulation of transcription | [14](http://bioinfo.cau.edu.cn/agriGO/termDetail.php?session=841659544&GO=GO:0045449) | 231 | 1.20E-09 | 3.90E-08 |
| GO:0006091 | P | generation of precursor metabolites and energy | [8](http://bioinfo.cau.edu.cn/agriGO/termDetail.php?session=841659544&GO=GO:0006091) | 49 | 1.60E-09 | 4.90E-08 |
| GO:0010556 | P | regulation of macromolecule biosynthetic process | [14](http://bioinfo.cau.edu.cn/agriGO/termDetail.php?session=841659544&GO=GO:0010556) | 238 | 1.80E-09 | 5.10E-08 |
| GO:0044249 | P | cellular biosynthetic process | [24](http://bioinfo.cau.edu.cn/agriGO/termDetail.php?session=841659544&GO=GO:0044249) | 760 | 1.90E-09 | 5.10E-08 |
| GO:0019219 | P | regulation of nucleobase, nucleoside, nucleotide and nucleic acid metabolic process | [14](http://bioinfo.cau.edu.cn/agriGO/termDetail.php?session=841659544&GO=GO:0019219) | 239 | 1.90E-09 | 5.10E-08 |
| GO:0051171 | P | regulation of nitrogen compound metabolic process | [14](http://bioinfo.cau.edu.cn/agriGO/termDetail.php?session=841659544&GO=GO:0051171) | 244 | 2.50E-09 | 6.30E-08 |
| GO:0009889 | P | regulation of biosynthetic process | [14](http://bioinfo.cau.edu.cn/agriGO/termDetail.php?session=841659544&GO=GO:0009889) | 248 | 3.10E-09 | 7.10E-08 |
| GO:0031326 | P | regulation of cellular biosynthetic process | [14](http://bioinfo.cau.edu.cn/agriGO/termDetail.php?session=841659544&GO=GO:0031326) | 248 | 3.10E-09 | 7.10E-08 |
| GO:0080090 | P | regulation of primary metabolic process | [14](http://bioinfo.cau.edu.cn/agriGO/termDetail.php?session=841659544&GO=GO:0080090) | 256 | 4.60E-09 | 1.00E-07 |
| GO:0006350 | P | transcription | [14](http://bioinfo.cau.edu.cn/agriGO/termDetail.php?session=841659544&GO=GO:0006350) | 262 | 6.10E-09 | 1.30E-07 |
| GO:0031323 | P | regulation of cellular metabolic process | [14](http://bioinfo.cau.edu.cn/agriGO/termDetail.php?session=841659544&GO=GO:0031323) | 268 | 8.20E-09 | 1.70E-07 |
| GO:0010468 | P | regulation of gene expression | [14](http://bioinfo.cau.edu.cn/agriGO/termDetail.php?session=841659544&GO=GO:0010468) | 275 | 1.10E-08 | 2.30E-07 |
| GO:0060255 | P | regulation of macromolecule metabolic process | [14](http://bioinfo.cau.edu.cn/agriGO/termDetail.php?session=841659544&GO=GO:0060255) | 284 | 1.70E-08 | 3.30E-07 |
| GO:0010035 | P | response to inorganic substance | [6](http://bioinfo.cau.edu.cn/agriGO/termDetail.php?session=841659544&GO=GO:0010035) | 27 | 2.50E-08 | 4.80E-07 |
| GO:0009617 | P | response to bacterium | [6](http://bioinfo.cau.edu.cn/agriGO/termDetail.php?session=841659544&GO=GO:0009617) | 29 | 4.00E-08 | 7.30E-07 |
| GO:0019222 | P | regulation of metabolic process | [14](http://bioinfo.cau.edu.cn/agriGO/termDetail.php?session=841659544&GO=GO:0019222) | 309 | 4.80E-08 | 8.60E-07 |
| GO:0009611 | P | response to wounding | [6](http://bioinfo.cau.edu.cn/agriGO/termDetail.php?session=841659544&GO=GO:0009611) | 30 | 5.00E-08 | 8.60E-07 |
| GO:0019538 | P | protein metabolic process | [15](http://bioinfo.cau.edu.cn/agriGO/termDetail.php?session=841659544&GO=GO:0019538) | 374 | 8.10E-08 | 1.40E-06 |
| GO:0010876 | P | lipid localization | [5](http://bioinfo.cau.edu.cn/agriGO/termDetail.php?session=841659544&GO=GO:0010876) | 18 | 1.10E-07 | 1.80E-06 |
| GO:0009639 | P | response to red or far red light | [8](http://bioinfo.cau.edu.cn/agriGO/termDetail.php?session=841659544&GO=GO:0009639) | 83 | 1.10E-07 | 1.80E-06 |
| GO:0006807 | P | nitrogen compound metabolic process | [20](http://bioinfo.cau.edu.cn/agriGO/termDetail.php?session=841659544&GO=GO:0006807) | 702 | 1.90E-07 | 2.90E-06 |
| GO:0009059 | P | macromolecule biosynthetic process | [16](http://bioinfo.cau.edu.cn/agriGO/termDetail.php?session=841659544&GO=GO:0009059) | 485 | 4.20E-07 | 6.40E-06 |
| GO:0019684 | P | photosynthesis, light reaction | [7](http://bioinfo.cau.edu.cn/agriGO/termDetail.php?session=841659544&GO=GO:0019684) | 78 | 1.10E-06 | 1.70E-05 |
| GO:0006970 | P | response to osmotic stress | [7](http://bioinfo.cau.edu.cn/agriGO/termDetail.php?session=841659544&GO=GO:0006970) | 81 | 1.50E-06 | 2.10E-05 |
| GO:0034645 | P | cellular macromolecule biosynthetic process | [15](http://bioinfo.cau.edu.cn/agriGO/termDetail.php?session=841659544&GO=GO:0034645) | 482 | 1.90E-06 | 2.70E-05 |
| GO:0051188 | P | cofactor biosynthetic process | [5](http://bioinfo.cau.edu.cn/agriGO/termDetail.php?session=841659544&GO=GO:0051188) | 31 | 2.10E-06 | 2.80E-05 |
| GO:0050790 | P | regulation of catalytic activity | [5](http://bioinfo.cau.edu.cn/agriGO/termDetail.php?session=841659544&GO=GO:0050790) | 31 | 2.10E-06 | 2.80E-05 |
| GO:0055114 | P | oxidation reduction | [5](http://bioinfo.cau.edu.cn/agriGO/termDetail.php?session=841659544&GO=GO:0055114) | 32 | 2.40E-06 | 3.10E-05 |
| GO:0065009 | P | regulation of molecular function | [5](http://bioinfo.cau.edu.cn/agriGO/termDetail.php?session=841659544&GO=GO:0065009) | 32 | 2.40E-06 | 3.10E-05 |
| GO:0006139 | P | nucleobase, nucleoside, nucleotide and nucleic acid metabolic process | [16](http://bioinfo.cau.edu.cn/agriGO/termDetail.php?session=841659544&GO=GO:0006139) | 562 | 2.80E-06 | 3.50E-05 |
| GO:0034641 | P | cellular nitrogen compound metabolic process | [7](http://bioinfo.cau.edu.cn/agriGO/termDetail.php?session=841659544&GO=GO:0034641) | 93 | 3.70E-06 | 4.60E-05 |
| GO:0006457 | P | protein folding | [5](http://bioinfo.cau.edu.cn/agriGO/termDetail.php?session=841659544&GO=GO:0006457) | 36 | 4.40E-06 | 5.30E-05 |
| GO:0043687 | P | post-translational protein modification | [5](http://bioinfo.cau.edu.cn/agriGO/termDetail.php?session=841659544&GO=GO:0043687) | 36 | 4.40E-06 | 5.30E-05 |
| GO:0048518 | P | positive regulation of biological process | [5](http://bioinfo.cau.edu.cn/agriGO/termDetail.php?session=841659544&GO=GO:0048518) | 41 | 8.60E-06 | 0.0001 |
| GO:0010467 | P | gene expression | [15](http://bioinfo.cau.edu.cn/agriGO/termDetail.php?session=841659544&GO=GO:0010467) | 575 | 1.50E-05 | 0.00017 |
| GO:0009637 | P | response to blue light | [5](http://bioinfo.cau.edu.cn/agriGO/termDetail.php?session=841659544&GO=GO:0009637) | 49 | 2.10E-05 | 0.00023 |
| GO:0046148 | P | pigment biosynthetic process | [6](http://bioinfo.cau.edu.cn/agriGO/termDetail.php?session=841659544&GO=GO:0046148) | 83 | 2.30E-05 | 0.00025 |
| GO:0032501 | P | multicellular organismal process | [14](http://bioinfo.cau.edu.cn/agriGO/termDetail.php?session=841659544&GO=GO:0032501) | 527 | 2.30E-05 | 0.00025 |
| GO:0044267 | P | cellular protein metabolic process | [11](http://bioinfo.cau.edu.cn/agriGO/termDetail.php?session=841659544&GO=GO:0044267) | 338 | 2.70E-05 | 0.00029 |
| GO:0051186 | P | cofactor metabolic process | [5](http://bioinfo.cau.edu.cn/agriGO/termDetail.php?session=841659544&GO=GO:0051186) | 52 | 2.80E-05 | 0.00029 |
| GO:0010114 | P | response to red light | [5](http://bioinfo.cau.edu.cn/agriGO/termDetail.php?session=841659544&GO=GO:0010114) | 54 | 3.40E-05 | 0.00035 |
| GO:0016070 | P | RNA metabolic process | [9](http://bioinfo.cau.edu.cn/agriGO/termDetail.php?session=841659544&GO=GO:0016070) | 232 | 3.60E-05 | 0.00037 |
| GO:0006979 | P | response to oxidative stress | [6](http://bioinfo.cau.edu.cn/agriGO/termDetail.php?session=841659544&GO=GO:0006979) | 94 | 4.70E-05 | 0.00046 |
| GO:0008610 | P | lipid biosynthetic process | [6](http://bioinfo.cau.edu.cn/agriGO/termDetail.php?session=841659544&GO=GO:0008610) | 94 | 4.70E-05 | 0.00046 |
| GO:0009607 | P | response to biotic stimulus | [13](http://bioinfo.cau.edu.cn/agriGO/termDetail.php?session=841659544&GO=GO:0009607) | 497 | 5.10E-05 | 0.00049 |
| GO:0042440 | P | pigment metabolic process | [6](http://bioinfo.cau.edu.cn/agriGO/termDetail.php?session=841659544&GO=GO:0042440) | 99 | 6.30E-05 | 0.00059 |
| GO:0009416 | P | response to light stimulus | [12](http://bioinfo.cau.edu.cn/agriGO/termDetail.php?session=841659544&GO=GO:0009416) | 439 | 6.40E-05 | 0.0006 |
| GO:0009743 | P | response to carbohydrate stimulus | [5](http://bioinfo.cau.edu.cn/agriGO/termDetail.php?session=841659544&GO=GO:0009743) | 62 | 6.60E-05 | 0.0006 |
| GO:0009314 | P | response to radiation | [12](http://bioinfo.cau.edu.cn/agriGO/termDetail.php?session=841659544&GO=GO:0009314) | 453 | 8.60E-05 | 0.00078 |
| GO:0009651 | P | response to salt stress | [5](http://bioinfo.cau.edu.cn/agriGO/termDetail.php?session=841659544&GO=GO:0009651) | 70 | 0.00012 | 0.001 |
| GO:0044271 | P | cellular nitrogen compound biosynthetic process | [5](http://bioinfo.cau.edu.cn/agriGO/termDetail.php?session=841659544&GO=GO:0044271) | 76 | 0.00017 | 0.0015 |
| GO:0032502 | P | developmental process | [13](http://bioinfo.cau.edu.cn/agriGO/termDetail.php?session=841659544&GO=GO:0032502) | 574 | 0.00021 | 0.0018 |
| GO:0009620 | P | response to fungus | [6](http://bioinfo.cau.edu.cn/agriGO/termDetail.php?session=841659544&GO=GO:0009620) | 124 | 0.00022 | 0.0018 |
| GO:0032787 | P | monocarboxylic acid metabolic process | [5](http://bioinfo.cau.edu.cn/agriGO/termDetail.php?session=841659544&GO=GO:0032787) | 85 | 0.00029 | 0.0025 |
| GO:0051704 | P | multi-organism process | [13](http://bioinfo.cau.edu.cn/agriGO/termDetail.php?session=841659544&GO=GO:0051704) | 605 | 0.00034 | 0.0028 |
| GO:0033036 | P | macromolecule localization | [5](http://bioinfo.cau.edu.cn/agriGO/termDetail.php?session=841659544&GO=GO:0033036) | 90 | 0.00038 | 0.0031 |
| GO:0006869 | P | lipid transport | [5](http://bioinfo.cau.edu.cn/agriGO/termDetail.php?session=841659544&GO=GO:0006869) | 94 | 0.00047 | 0.0038 |
| GO:0007275 | P | multicellular organismal development | [11](http://bioinfo.cau.edu.cn/agriGO/termDetail.php?session=841659544&GO=GO:0007275) | 507 | 0.00086 | 0.0068 |
| GO:0006810 | P | transport | [11](http://bioinfo.cau.edu.cn/agriGO/termDetail.php?session=841659544&GO=GO:0006810) | 540 | 0.0014 | 0.011 |
| GO:0043412 | P | macromolecule modification | [6](http://bioinfo.cau.edu.cn/agriGO/termDetail.php?session=841659544&GO=GO:0043412) | 180 | 0.0015 | 0.012 |
| GO:0051234 | P | establishment of localization | [11](http://bioinfo.cau.edu.cn/agriGO/termDetail.php?session=841659544&GO=GO:0051234) | 545 | 0.0015 | 0.012 |
| GO:0051179 | P | localization | [11](http://bioinfo.cau.edu.cn/agriGO/termDetail.php?session=841659544&GO=GO:0051179) | 569 | 0.0021 | 0.016 |
| GO:0048856 | P | anatomical structure development | [8](http://bioinfo.cau.edu.cn/agriGO/termDetail.php?session=841659544&GO=GO:0048856) | 330 | 0.0021 | 0.016 |
| GO:0006464 | P | protein modification process | [5](http://bioinfo.cau.edu.cn/agriGO/termDetail.php?session=841659544&GO=GO:0006464) | 144 | 0.0031 | 0.023 |
| GO:0003700 | F | transcription factor activity | [18](http://bioinfo.cau.edu.cn/agriGO/termDetail.php?session=841659544&GO=GO:0003700) | 46 | 3.40E-27 | 6.90E-25 |
| GO:0046906 | F | tetrapyrrole binding | [6](http://bioinfo.cau.edu.cn/agriGO/termDetail.php?session=841659544&GO=GO:0046906) | 6 | 9.60E-14 | 9.80E-12 |
| GO:0016491 | F | oxidoreductase activity | [17](http://bioinfo.cau.edu.cn/agriGO/termDetail.php?session=841659544&GO=GO:0016491) | 555 | 5.30E-07 | 3.60E-05 |
| GO:0016168 | F | chlorophyll binding | [5](http://bioinfo.cau.edu.cn/agriGO/termDetail.php?session=841659544&GO=GO:0016168) | 32 | 2.40E-06 | 0.00012 |
| GO:0008289 | F | lipid binding | [5](http://bioinfo.cau.edu.cn/agriGO/termDetail.php?session=841659544&GO=GO:0008289) | 45 | 1.40E-05 | 0.00056 |
| GO:0043169 | F | cation binding | [10](http://bioinfo.cau.edu.cn/agriGO/termDetail.php?session=841659544&GO=GO:0043169) | 306 | 5.90E-05 | 0.0015 |
| GO:0043167 | F | ion binding | [10](http://bioinfo.cau.edu.cn/agriGO/termDetail.php?session=841659544&GO=GO:0043167) | 306 | 5.90E-05 | 0.0015 |
| GO:0046872 | F | metal ion binding | [10](http://bioinfo.cau.edu.cn/agriGO/termDetail.php?session=841659544&GO=GO:0046872) | 297 | 4.60E-05 | 0.0015 |
| GO:0016740 | F | transferase activity | [17](http://bioinfo.cau.edu.cn/agriGO/termDetail.php?session=841659544&GO=GO:0016740) | 902 | 0.00023 | 0.0046 |
| GO:0016829 | F | lyase activity | [5](http://bioinfo.cau.edu.cn/agriGO/termDetail.php?session=841659544&GO=GO:0016829) | 82 | 0.00025 | 0.0046 |
| GO:0016853 | F | isomerase activity | [5](http://bioinfo.cau.edu.cn/agriGO/termDetail.php?session=841659544&GO=GO:0016853) | 80 | 0.00022 | 0.0046 |
| GO:0060089 | F | molecular transducer activity | [5](http://bioinfo.cau.edu.cn/agriGO/termDetail.php?session=841659544&GO=GO:0060089) | 90 | 0.00038 | 0.006 |
| GO:0004871 | F | signal transducer activity | [5](http://bioinfo.cau.edu.cn/agriGO/termDetail.php?session=841659544&GO=GO:0004871) | 90 | 0.00038 | 0.006 |
| GO:0046914 | F | transition metal ion binding | [7](http://bioinfo.cau.edu.cn/agriGO/termDetail.php?session=841659544&GO=GO:0046914) | 195 | 0.00041 | 0.006 |
| GO:0005515 | F | protein binding | [15](http://bioinfo.cau.edu.cn/agriGO/termDetail.php?session=841659544&GO=GO:0005515) | 955 | 0.0029 | 0.039 |
| GO:0004888 | F | transmembrane receptor activity | [5](http://bioinfo.cau.edu.cn/agriGO/termDetail.php?session=841659544&GO=GO:0004888) | 151 | 0.0038 | 0.048 |
| GO:0009579 | C | thylakoid | [22](http://bioinfo.cau.edu.cn/agriGO/termDetail.php?session=841659544&GO=GO:0009579) | 52 | 9.10E-34 | 1.80E-31 |
| GO:0044436 | C | thylakoid part | [17](http://bioinfo.cau.edu.cn/agriGO/termDetail.php?session=841659544&GO=GO:0044436) | 46 | 2.90E-25 | 2.90E-23 |
| GO:0031976 | C | plastid thylakoid | [16](http://bioinfo.cau.edu.cn/agriGO/termDetail.php?session=841659544&GO=GO:0031976) | 41 | 2.40E-24 | 1.20E-22 |
| GO:0009534 | C | chloroplast thylakoid | [16](http://bioinfo.cau.edu.cn/agriGO/termDetail.php?session=841659544&GO=GO:0009534) | 41 | 2.40E-24 | 1.20E-22 |
| GO:0031984 | C | organelle subcompartment | [16](http://bioinfo.cau.edu.cn/agriGO/termDetail.php?session=841659544&GO=GO:0031984) | 42 | 3.90E-24 | 1.50E-22 |
| GO:0044422 | C | organelle part | [35](http://bioinfo.cau.edu.cn/agriGO/termDetail.php?session=841659544&GO=GO:0044422) | 523 | 1.20E-22 | 3.40E-21 |
| GO:0044446 | C | intracellular organelle part | [35](http://bioinfo.cau.edu.cn/agriGO/termDetail.php?session=841659544&GO=GO:0044446) | 523 | 1.20E-22 | 3.40E-21 |
| GO:0044464 | C | cell part | [121](http://bioinfo.cau.edu.cn/agriGO/termDetail.php?session=841659544&GO=GO:0044464) | 6952 | 9.60E-16 | 2.10E-14 |
| GO:0005623 | C | cell | [121](http://bioinfo.cau.edu.cn/agriGO/termDetail.php?session=841659544&GO=GO:0005623) | 6952 | 9.60E-16 | 2.10E-14 |
| GO:0044435 | C | plastid part | [31](http://bioinfo.cau.edu.cn/agriGO/termDetail.php?session=841659544&GO=GO:0044435) | 718 | 4.40E-15 | 8.70E-14 |
| GO:0044434 | C | chloroplast part | [29](http://bioinfo.cau.edu.cn/agriGO/termDetail.php?session=841659544&GO=GO:0044434) | 625 | 4.90E-15 | 8.90E-14 |
| GO:0034357 | C | photosynthetic membrane | [19](http://bioinfo.cau.edu.cn/agriGO/termDetail.php?session=841659544&GO=GO:0034357) | 239 | 1.40E-14 | 2.30E-13 |
| GO:0030076 | C | light-harvesting complex | [5](http://bioinfo.cau.edu.cn/agriGO/termDetail.php?session=841659544&GO=GO:0030076) | 5 | 1.40E-11 | 2.10E-10 |
| GO:0043234 | C | protein complex | [17](http://bioinfo.cau.edu.cn/agriGO/termDetail.php?session=841659544&GO=GO:0043234) | 319 | 1.70E-10 | 2.50E-09 |
| GO:0009521 | C | photosystem | [5](http://bioinfo.cau.edu.cn/agriGO/termDetail.php?session=841659544&GO=GO:0009521) | 7 | 2.90E-10 | 3.40E-09 |
| GO:0009507 | C | chloroplast | [48](http://bioinfo.cau.edu.cn/agriGO/termDetail.php?session=841659544&GO=GO:0009507) | 2335 | 2.80E-10 | 3.40E-09 |
| GO:0009526 | C | plastid envelope | [8](http://bioinfo.cau.edu.cn/agriGO/termDetail.php?session=841659544&GO=GO:0009526) | 40 | 2.90E-10 | 3.40E-09 |
| GO:0009536 | C | plastid | [50](http://bioinfo.cau.edu.cn/agriGO/termDetail.php?session=841659544&GO=GO:0009536) | 2507 | 3.40E-10 | 3.80E-09 |
| GO:0044425 | C | membrane part | [17](http://bioinfo.cau.edu.cn/agriGO/termDetail.php?session=841659544&GO=GO:0044425) | 364 | 1.30E-09 | 1.30E-08 |
| GO:0009941 | C | chloroplast envelope | [7](http://bioinfo.cau.edu.cn/agriGO/termDetail.php?session=841659544&GO=GO:0009941) | 33 | 2.40E-09 | 2.40E-08 |
| GO:0031975 | C | envelope | [9](http://bioinfo.cau.edu.cn/agriGO/termDetail.php?session=841659544&GO=GO:0031975) | 84 | 7.30E-09 | 6.60E-08 |
| GO:0031967 | C | organelle envelope | [9](http://bioinfo.cau.edu.cn/agriGO/termDetail.php?session=841659544&GO=GO:0031967) | 84 | 7.30E-09 | 6.60E-08 |
| GO:0031090 | C | organelle membrane | [19](http://bioinfo.cau.edu.cn/agriGO/termDetail.php?session=841659544&GO=GO:0031090) | 658 | 3.00E-07 | 2.60E-06 |
| GO:0005634 | C | nucleus | [11](http://bioinfo.cau.edu.cn/agriGO/termDetail.php?session=841659544&GO=GO:0005634) | 212 | 3.20E-07 | 2.60E-06 |
| GO:0032991 | C | macromolecular complex | [17](http://bioinfo.cau.edu.cn/agriGO/termDetail.php?session=841659544&GO=GO:0032991) | 601 | 1.50E-06 | 1.20E-05 |
| GO:0009570 | C | chloroplast stroma | [5](http://bioinfo.cau.edu.cn/agriGO/termDetail.php?session=841659544&GO=GO:0009570) | 31 | 2.10E-06 | 1.60E-05 |
| GO:0009532 | C | plastid stroma | [6](http://bioinfo.cau.edu.cn/agriGO/termDetail.php?session=841659544&GO=GO:0009532) | 60 | 3.50E-06 | 2.60E-05 |
| GO:0005773 | C | vacuole | [5](http://bioinfo.cau.edu.cn/agriGO/termDetail.php?session=841659544&GO=GO:0005773) | 42 | 9.70E-06 | 6.80E-05 |
| GO:0044444 | C | cytoplasmic part | [61](http://bioinfo.cau.edu.cn/agriGO/termDetail.php?session=841659544&GO=GO:0044444) | 5113 | 9.00E-05 | 0.00059 |
| GO:0010287 | C | plastoglobule | [5](http://bioinfo.cau.edu.cn/agriGO/termDetail.php?session=841659544&GO=GO:0010287) | 66 | 8.90E-05 | 0.00059 |
| GO:0005737 | C | cytoplasm | [63](http://bioinfo.cau.edu.cn/agriGO/termDetail.php?session=841659544&GO=GO:0005737) | 5501 | 0.0002 | 0.0013 |
| GO:0044424 | C | intracellular part | [80](http://bioinfo.cau.edu.cn/agriGO/termDetail.php?session=841659544&GO=GO:0044424) | 7433 | 0.00026 | 0.0016 |
| GO:0016020 | C | membrane | [43](http://bioinfo.cau.edu.cn/agriGO/termDetail.php?session=841659544&GO=GO:0016020) | 3495 | 0.00037 | 0.0022 |
| GO:0043231 | C | intracellular membrane-bounded organelle | [67](http://bioinfo.cau.edu.cn/agriGO/termDetail.php?session=841659544&GO=GO:0043231) | 6132 | 0.00043 | 0.0025 |
| GO:0043227 | C | membrane-bounded organelle | [67](http://bioinfo.cau.edu.cn/agriGO/termDetail.php?session=841659544&GO=GO:0043227) | 6138 | 0.00044 | 0.0025 |
| GO:0005622 | C | intracellular | [81](http://bioinfo.cau.edu.cn/agriGO/termDetail.php?session=841659544&GO=GO:0005622) | 7736 | 0.00049 | 0.0027 |
| GO:0043229 | C | intracellular organelle | [67](http://bioinfo.cau.edu.cn/agriGO/termDetail.php?session=841659544&GO=GO:0043229) | 6542 | 0.0018 | 0.0097 |
| GO:0043226 | C | organelle | [67](http://bioinfo.cau.edu.cn/agriGO/termDetail.php?session=841659544&GO=GO:0043226) | 6546 | 0.0019 | 0.0097 |
| GO:0048046 | C | apoplast | [5](http://bioinfo.cau.edu.cn/agriGO/termDetail.php?session=841659544&GO=GO:0048046) | 173 | 0.0067 | 0.034 |
| GO:0005576 | C | extracellular region | [6](http://bioinfo.cau.edu.cn/agriGO/termDetail.php?session=841659544&GO=GO:0005576) | 255 | 0.0082 | 0.041 |

**P**: Biological Process; **C**: Cellular Component; **F**: Mollecular Function; **Number in input list**: Gene number of the GO term from 213 differential expression genes; **Number in BG/Ref**: Gene number of the GO term from all genes in Arabidopsis thaliana; **FDR**: false discovery rate.
