# Supplementary material for: Disruption of the Homogentisate Solanesyltransferase Gene Results in Albino and Dwarf Phenotypes and Root, Trichome and Stomata Defects in Arabidopsis thaliana
Source: PLoS One. 2014 Apr 17;9(4):e94031. doi: 10.1371/journal.pone.0094031 (PMC3990575; doi:10.1371/journal.pone.0094031)
Supplement: Table S4 — GO terms in Sub-Cellular Structure/Components generated by PAGE. (DOC) [file pone.0094031.s011.doc]

**Table S4** GO terms in Sub-Cellular Structure/Components generated by PAGE.

| **GO term** | **Description** | **Number** | **Z-Score** | **FDR** |
| --- | --- | --- | --- | --- |
| GO:0034357 | photosynthetic membrane | [28](http://bioinfo.cau.edu.cn/agriGO/PAGEtermDetail.php?session=905980218&GO=GO:0034357) | -2.6 | 0.025 |
| GO:0044436 | thylakoid part | [30](http://bioinfo.cau.edu.cn/agriGO/PAGEtermDetail.php?session=905980218&GO=GO:0044436) | -2.7 | 0.025 |
| GO:0031976 | plastid thylakoid | [33](http://bioinfo.cau.edu.cn/agriGO/PAGEtermDetail.php?session=905980218&GO=GO:0031976) | -2.8 | 0.025 |
| GO:0044422 | organelle part | [54](http://bioinfo.cau.edu.cn/agriGO/PAGEtermDetail.php?session=905980218&GO=GO:0044422) | -2.6 | 0.025 |
| GO:0009941 | chloroplast envelope | [20](http://bioinfo.cau.edu.cn/agriGO/PAGEtermDetail.php?session=905980218&GO=GO:0009941) | -2.5 | 0.026 |
| GO:0009526 | plastid envelope | [20](http://bioinfo.cau.edu.cn/agriGO/PAGEtermDetail.php?session=905980218&GO=GO:0009526) | -2.5 | 0.026 |
| GO:0016020 | membrane | [67](http://bioinfo.cau.edu.cn/agriGO/PAGEtermDetail.php?session=905980218&GO=GO:0016020) | -2.2 | 0.05 |
| GO:0055035 | plastid thylakoid membrane | [28](http://bioinfo.cau.edu.cn/agriGO/PAGEtermDetail.php?session=905980218&GO=GO:0055035) | -2.6 | 0.025 |
| GO:0043234 | protein complex | [19](http://bioinfo.cau.edu.cn/agriGO/PAGEtermDetail.php?session=905980218&GO=GO:0043234) | -2.8 | 0.025 |
| GO:0009579 | thylakoid | [34](http://bioinfo.cau.edu.cn/agriGO/PAGEtermDetail.php?session=905980218&GO=GO:0009579) | -3.1 | 0.018 |
| GO:0044435 | plastid part | [49](http://bioinfo.cau.edu.cn/agriGO/PAGEtermDetail.php?session=905980218&GO=GO:0044435) | -3.2 | 0.017 |
| GO:0044434 | chloroplast part | [49](http://bioinfo.cau.edu.cn/agriGO/PAGEtermDetail.php?session=905980218&GO=GO:0044434) | -3.2 | 0.017 |
| GO:0009507 | chloroplast | [80](http://bioinfo.cau.edu.cn/agriGO/PAGEtermDetail.php?session=905980218&GO=GO:0009507) | -4 | 0.0024 |
| GO:0042651 | thylakoid membrane | [28](http://bioinfo.cau.edu.cn/agriGO/PAGEtermDetail.php?session=905980218&GO=GO:0042651) | -2.6 | 0.025 |
| GO:0009535 | chloroplast thylakoid membrane | [28](http://bioinfo.cau.edu.cn/agriGO/PAGEtermDetail.php?session=905980218&GO=GO:0009535) | -2.6 | 0.025 |
| GO:0009534 | chloroplast thylakoid | [33](http://bioinfo.cau.edu.cn/agriGO/PAGEtermDetail.php?session=905980218&GO=GO:0009534) | -2.8 | 0.025 |
| GO:0009536 | plastid | [81](http://bioinfo.cau.edu.cn/agriGO/PAGEtermDetail.php?session=905980218&GO=GO:0009536) | -3.9 | 0.0027 |
| GO:0031975 | envelope | [21](http://bioinfo.cau.edu.cn/agriGO/PAGEtermDetail.php?session=905980218&GO=GO:0031975) | -2.5 | 0.026 |
| GO:0044446 | intracellular organelle part | [54](http://bioinfo.cau.edu.cn/agriGO/PAGEtermDetail.php?session=905980218&GO=GO:0044446) | -2.6 | 0.025 |
| GO:0044444 | cytoplasmic part | [103](http://bioinfo.cau.edu.cn/agriGO/PAGEtermDetail.php?session=905980218&GO=GO:0044444) | -2.5 | 0.029 |
| GO:0005634 | nucleus | [49](http://bioinfo.cau.edu.cn/agriGO/PAGEtermDetail.php?session=905980218&GO=GO:0005634) | 2.2 | 0.05 |
| GO:0031984 | organelle subcompartment | [33](http://bioinfo.cau.edu.cn/agriGO/PAGEtermDetail.php?session=905980218&GO=GO:0031984) | -2.8 | 0.025 |
| GO:0031967 | organelle envelope | [21](http://bioinfo.cau.edu.cn/agriGO/PAGEtermDetail.php?session=905980218&GO=GO:0031967) | -2.5 | 0.026 |
| GO:0032991 | macromolecular complex | [20](http://bioinfo.cau.edu.cn/agriGO/PAGEtermDetail.php?session=905980218&GO=GO:0032991) | -3 | 0.024 |

**Number**: Gene number in the GO term from differential expression genes submited. **FDR**: false discovery rate.
